# Kidney biopsy guidebook 2020 in Japan

**DOI:** 10.1007/s10157-020-01986-6

**Published:** 2021-02-19

**Authors:** Yoshifumi Ubara, Takehiko Kawaguchi, Tasuku Nagasawa, Kenichiro Miura, Takayuki Katsuno, Takashi Morikawa, Eiji Ishikawa, Masao Ogura, Hideki Matsumura, Ryota Kurayama, Shinsuke Matsumoto, Yuhji Marui, Shigeo Hara, Shoichi Maruyama, Ichiei Narita, Hirokazu Okada, Kazuhiko Tsuruya, Committee of Practical Guide for Kidney Biopsy 2020

**Affiliations:** 1 Department of Nephrology, Toranomon Hospital, Tokyo, Japan; 2 Department of Nephrology, National Hospital Organization Chibahigashi National Hospital, Chiba, Japan; 3 Division of Nephrology, Endocrinology and Vascular Medicine, Tohoku University Hospital, Sendai, Japan; 4 Department of Pediatric Nephrology, Tokyo Women’s Medical University, Tokyo, Japan; 5 Department of Nephrology and Rheumatology, Aichi Medical University, Aichi, Japan; 6 Department of Nephrology and Hypertension, Osaka City General Hospital, Osaka, Japan; 7 Department of Nephrology, Saiseikai Matsusaka General Hospital, Matsusaka, Mie Japan; 8 Department of Nephrology and Rheumatology, National Center for Child Health and Development, Tokyo, Japan; 9 Department of Pediatrics, Osaka Medical College, Osaka, Japan; 10 Department of Pediatrics, Kosei General Hospital, Tokyo, Japan; 11 Department of Pediatrics, Matsudo City General Hospital, Chiba, Japan; 12 Department of Urology, St. Marianna University School of Medicine, Kawasaki, Japan; 13 Department of Diagnostic Pathology, Kobe City Medical Center General Hospital, Kobe, Japan; 14 Department of Nephrology, Nagoya University, Nagoya, Japan; 15 Division of Clinical Nephrology and Rheumatology, Niigata University, Niigata, Japan; 16 Department of Nephrology, Saitama Medical University, Saitama, Japan; 17 Department of Nephrology, Nara Medical University, Nara, Japan; 18 Nephrology Center, Toranomon Hospital Kajigaya, 1-3-1, Kajigaya, Takatsu, Kawasaki, Kanagawa 212-8587 Japan

**Keywords:** Kidney biopsy, Indication of kidney biopsy in adults, Indication of kidney biopsy in children, Percutaneous native kidney biopsy under the ultrasonic guidance, Open (surgical) kidney biopsy and laparoscopic kidney biopsy, Relative contraindication for percutaneous native kidney biopsy, Bleeding complications after kidney biopsy

## Overview

A kidney biopsy is performed for a treatment strategy of renal disease by pathologically diagnosing renal disease. Kidney biopsy is a reliable gold standard technique, but various complications are common when obtaining tissue from an abundant vascular kidney. During a biopsy, vasovagal reflexes, including cold sweat, discomfort, nausea, vomiting, hypotension, and bradycardia, can occur. Hemorrhagic complications after a biopsy are important; 89% of hemorrhagic complications have been reported to occur within 24 h. Therefore, a cooperation system including nurses and physicians by performing intravenous feeding and medication, while performing electrocardiogram monitoring and oxygen saturation monitoring, is necessary.

Therefore, it is necessary to always take the benefits and risks of kidney biopsy into consideration and decide if there is an indication for kidney biopsy.

The conventional criteria for the indication of kidney biopsy for adults are shown in Table 1, according to previous reports [1–3]. However, there is an opinion that it is necessary to extend these indications [3]. The following opinions were sent by a member of the Japanese Society of Nephrology.

- There is an indication for kidney biopsy beyond the above indication. The indication must be considered in every case. It is important that it does not limit the experience-rich institutional practice.
- Nephrologists, including young doctors with little experience in kidney biopsy, should recognize the safety procedures that are necessary to prevent the threshold to high-risk clinical conditions from lowering.
- Cases of serious complications such as bleeding can happen, and the appropriate security guidelines for treatment should be prepared before a kidney biopsy.

**Table 1:** The conventional criteria for the indication of the kidney biopsy for adults

1. Glomerular hematuria with any degree of proteinuria
2. Isolated proteinuria > 1 g/day(or g/gCr)
3. Unexplained renal disease or intrinsic acute kidney injury
4. Renal manifestation related to systemic disease

The clinical treatment of renal disease is possible without performing a kidney biopsy. However, many nephrologists should note that a higher-quality clinical treatment is enabled by performing kidney biopsy.

The final decision of whether you perform kidney biopsy should be decided based on each institution’s guidelines and should be judged for every individual patient carefully. With respect to the decision, it is necessary to be performed based on the concept of “shared decision making: SDM,” after each attending physician explains the need and the risk of kidney biopsy to each patient thoroughly. We have provided explanations in the ‘Kidney biopsy guidebook 2020 in Japan’ along with questions and answers based on the results of a questionnaire survey for kidney biopsy that was performed in Japan from 2015 through 2017 by the Committee of Practical Guide for Kidney biopsy [4, 5], while adding the outline of the first edition of 2004 [1].

## Chapter 1: Indication for kidney biopsy (Table 2)

**Table 2:** Indication of kidney biopsy in adults

1. Isolated glomerular hematuria
2. Isolated proteinuria
3. Proteinuria and glomerular hematuria
4. Rapidly progressive glomerulonephritis
5. Intrinsic acute kidney injury
6. Systemic disease with a urinalysis abnormality
7. Systemic disease with renal dysfunction, and/or without urinalysis abnormality
8. Diabetes mellitus
9. Elderly renal disease
10. Hereditary renal disease
11. Repeated kidney biopsy


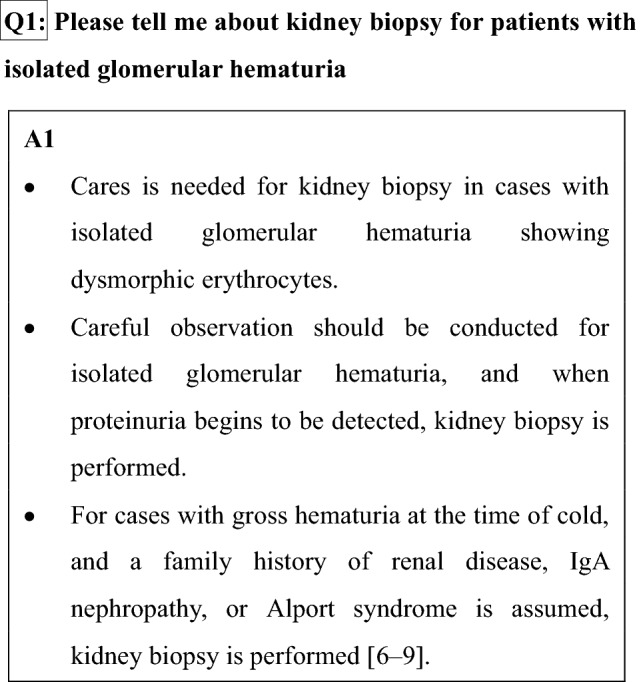



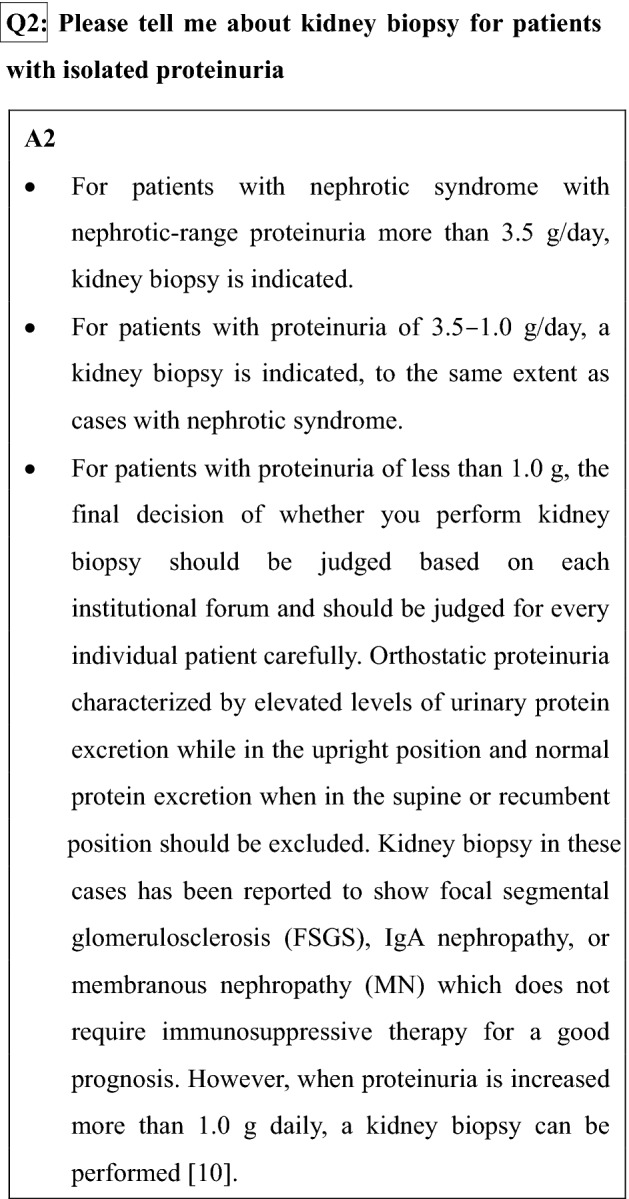



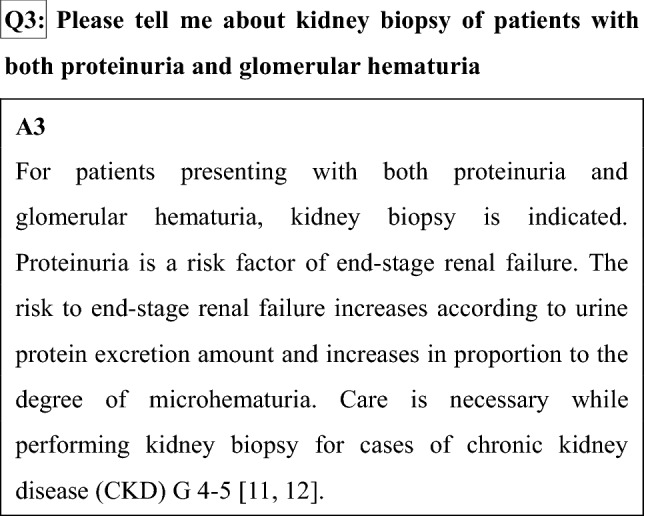



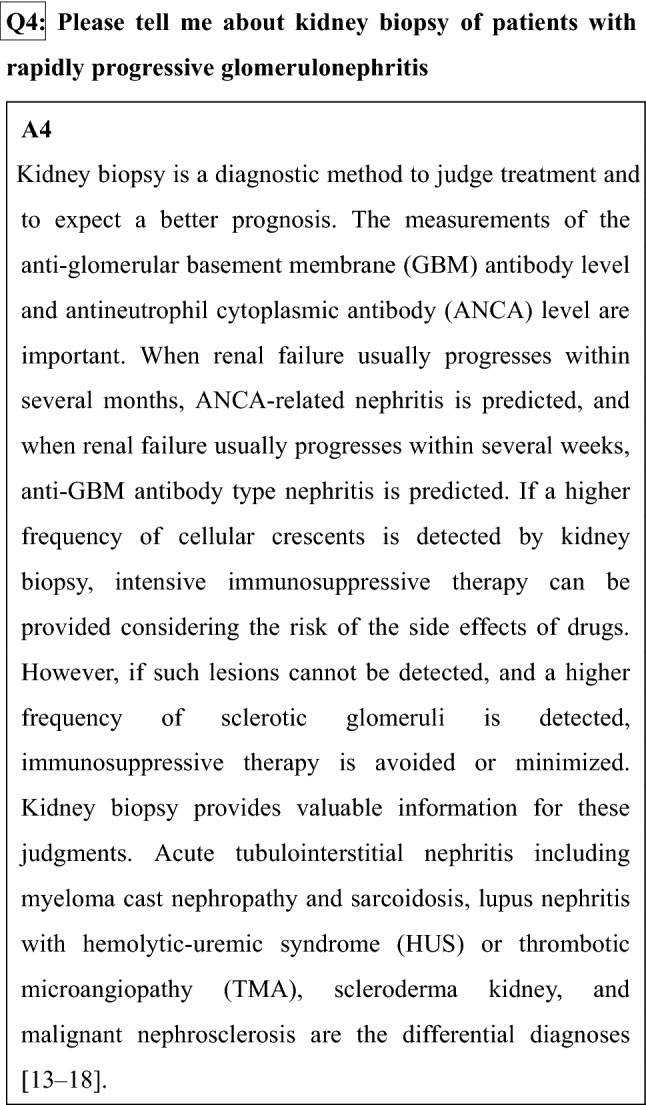



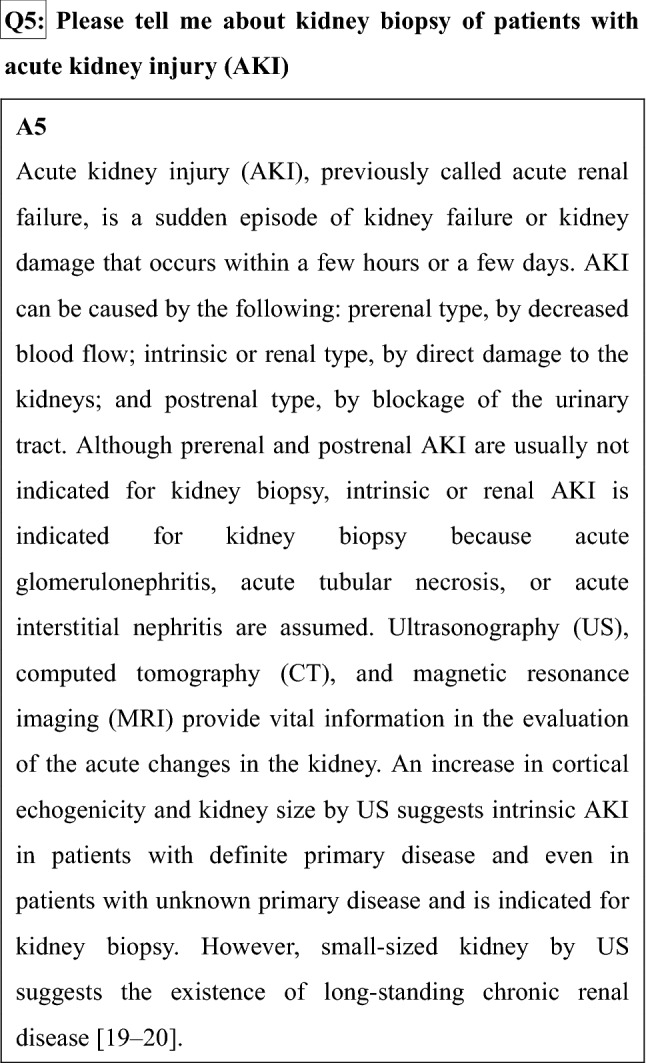



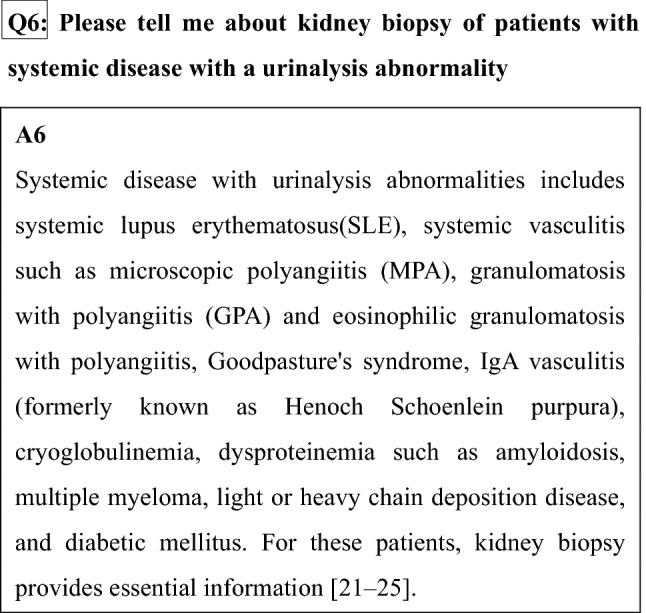



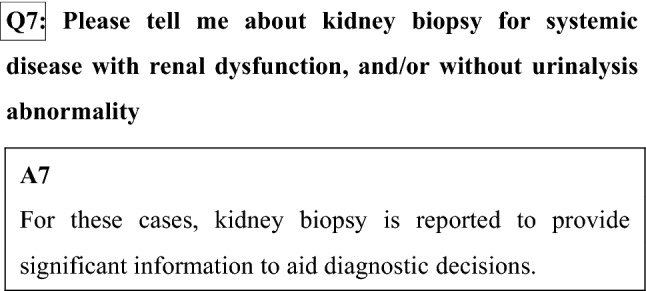

- Systemic disease with renal dysfunction, but without a urinalysis abnormality, includes acute or chronic tubulointerstitial nephritis secondary to sarcoidosis, drug-related disease such as tyrosine kinase inhibitors and checkpoint inhibitors. IgG4-related nephritis, or hypercalcemic nephropathy by activated cholecalciferol. A high value of tubular impairment markers such as β2-microglobulin (β2MG), α1-microglobulin (α1MG), or *N*-acetyl-β-d-glucosaminidase (NAG) is characteristic.
- Systemic lupus erythematosus without urinary abnormality is called silent lupus nephritis. Light microscopy of kidney biopsy is reported to show mild glomerular change with class I or class II on 74% of silent lupus nephritis according to ISN-RPS lupus nephritis classification, but immunofluorescent microscopy shows IgG and C1q stain, and electron microscopy shows electron-dense deposits in the mesangium or subepithelium, which are characteristic to lupus nephritis.
- Systemic vasculitis, including MPA, GPA and EGPA, can be diagnosed by extrarenal complications such as fever, upper respiratory tract disease, lung disease, neuropathy, and positivity for ANCA, even though urinary abnormality is negative. For these patients, kidney biopsy is reported to show crescent formation or vasculitis of small arteries with a frequency of 69%, although extrarenal organ biopsy may not show any vasculitis [26–29].

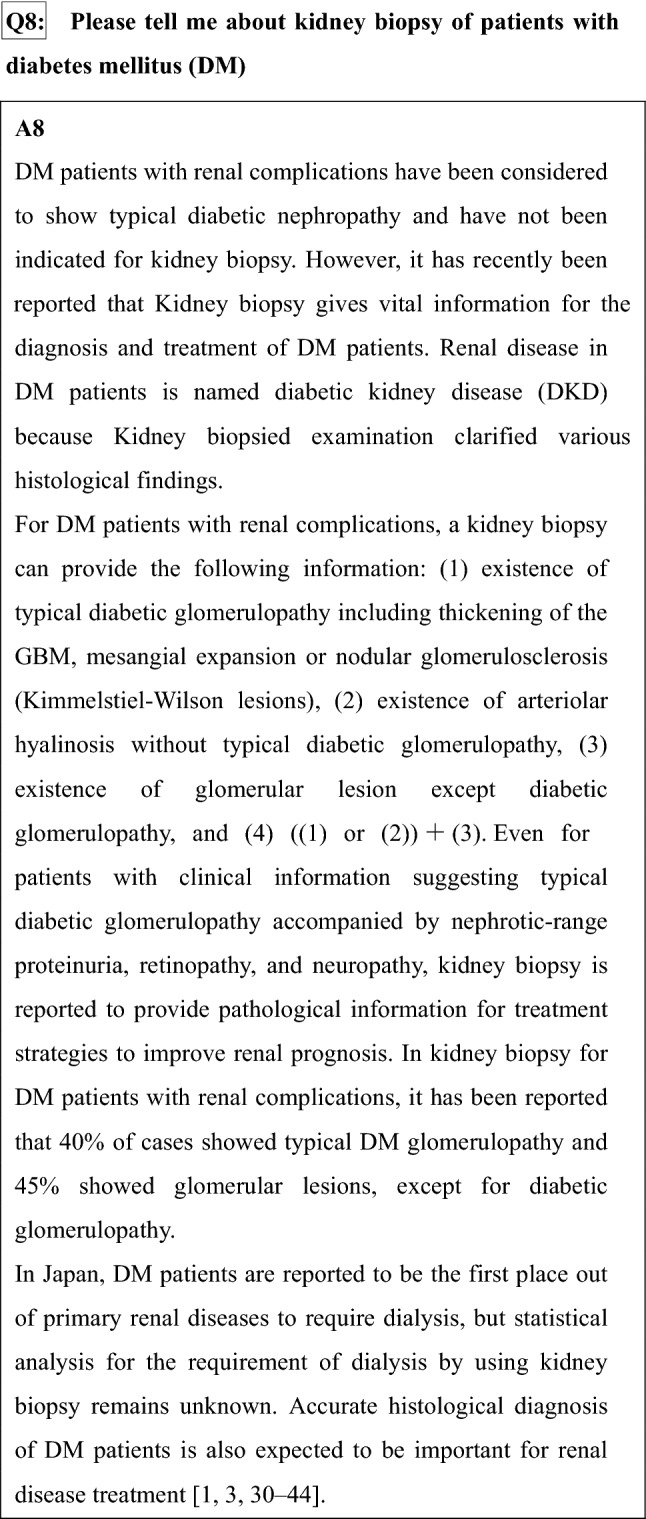



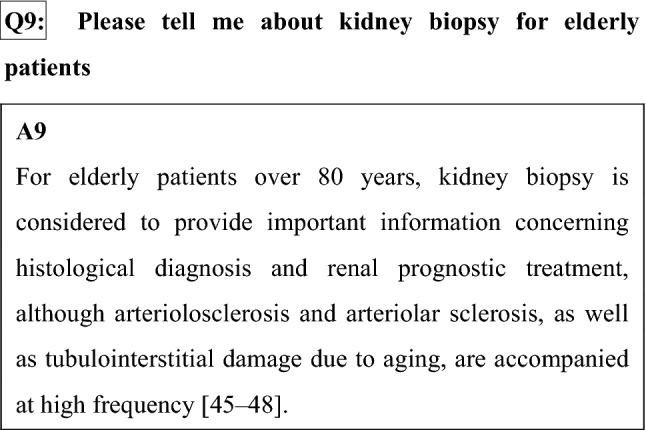



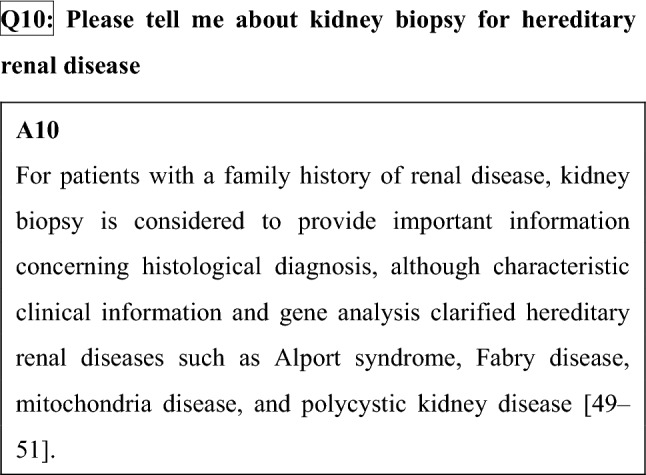



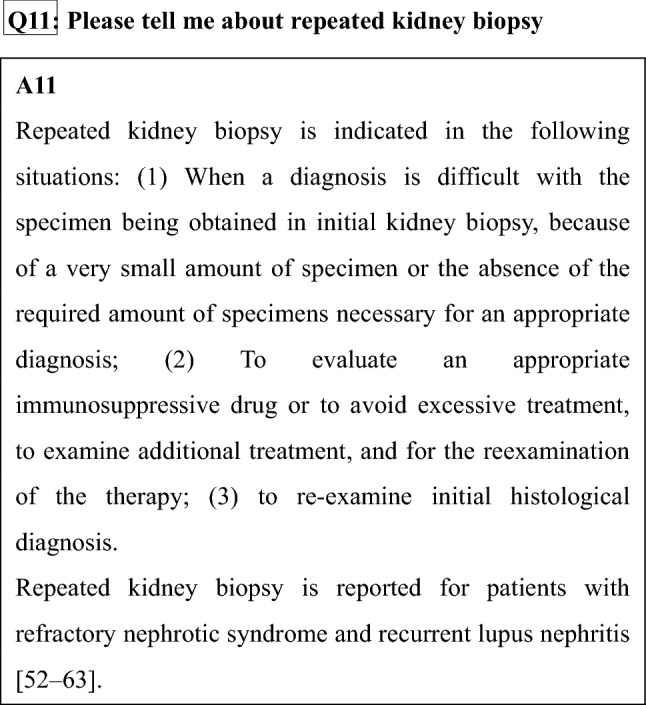


## Chapter 2: Kidney biopsy for patients with a clinical condition of high risk for percutaneous native kidney biopsy

### Overview

The following renal disease was contraindicated for percutaneous native kidney biopsy under the ultrasonic guidance in the previous edition of the guidebook because the risk of hemorrhagic complications after a kidney biopsy is very high, and renal tissue sampling necessary for diagnosis is not obtained [1] (Table 3). However, as biopsy techniques, by using a newer US device and automatic biopsy needle, improved safety, there have been several reported case series that required or enabled histological diagnosis by kidney biopsy [4]. Therefore, when the benefit is judged to exceed a risk, kidney biopsy is indicated for patients with a clinical condition of high risk. A kidney biopsy should be performed in institutions that can treat hemorrhagic complications. The following diseases are not absolute contraindicated anymore but are described as a renal disease with high risk by a question and answer method.

**Table 3:** Clinical condition of the high risk (equaling relative contraindication) for percutaneous native kidney biopsy under ultrasonic guidance

1. Solitary native kidney
2. Contracted kidneys, small hyperechoic kidneys or end-stage kidneys
3. Kidneys of anatomic abnormalities including horseshoe kidney, malrotation kidney and renal arterial aneurysm
4. Polycystic kidney disease
5. Hydronephrosis
6. Malignant nephrosclerosis related to hypertensive emergency and scleroderma renal crisis
7. Uncontrolled bleeding diathesis or severe thrombocytopenia
8. Pregnancy
9. Severe obesity
10. Renal mass including malignant neoplasma
11. Chronic anticoagulant therapy while taking antiplatelet or anticoagulant medication
12. Active renal or perirenal infection, or skin infection over the biopsy site
13. Inability to provide informed consent
14. Uncooperative patient or inability to follow instructions during biopsy


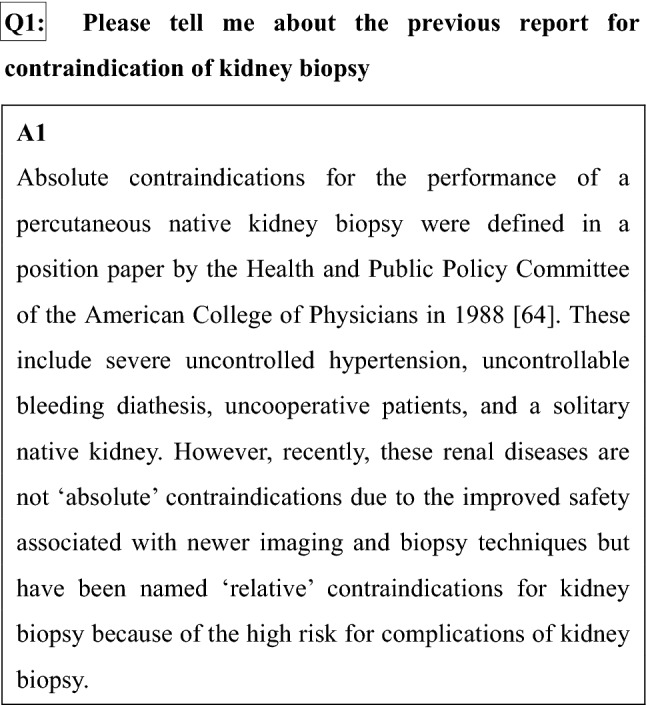



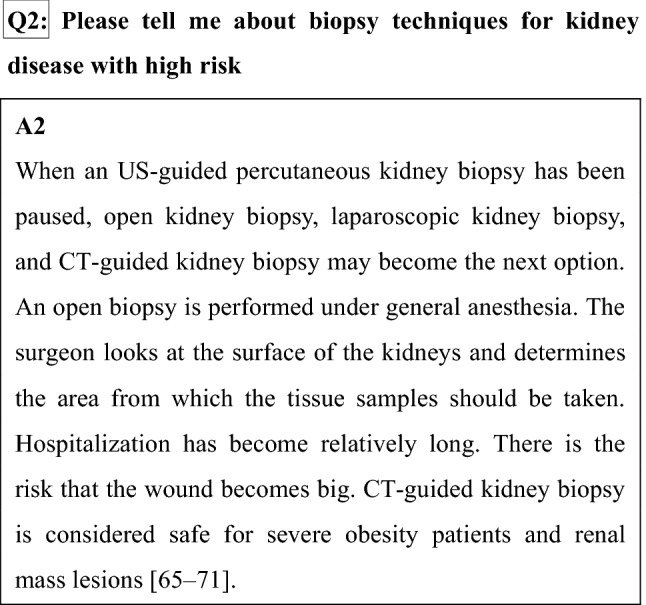



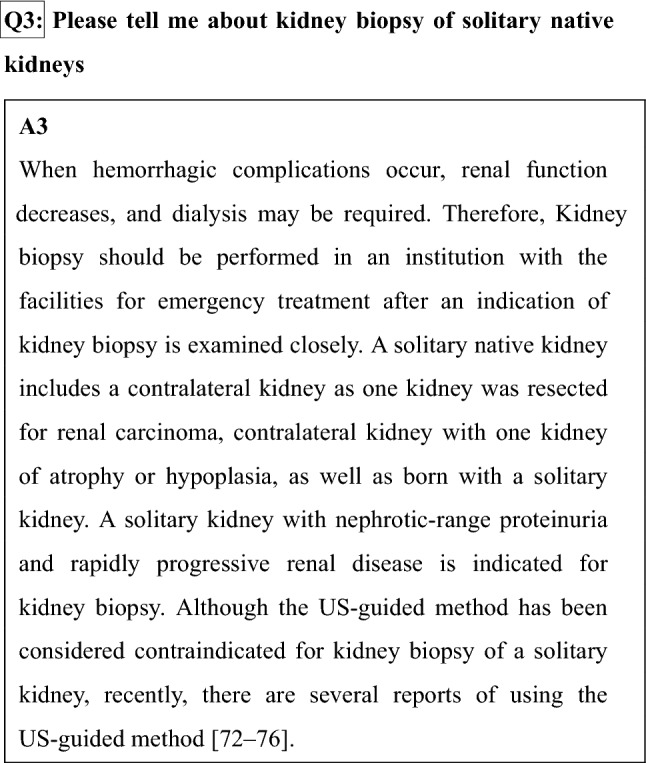



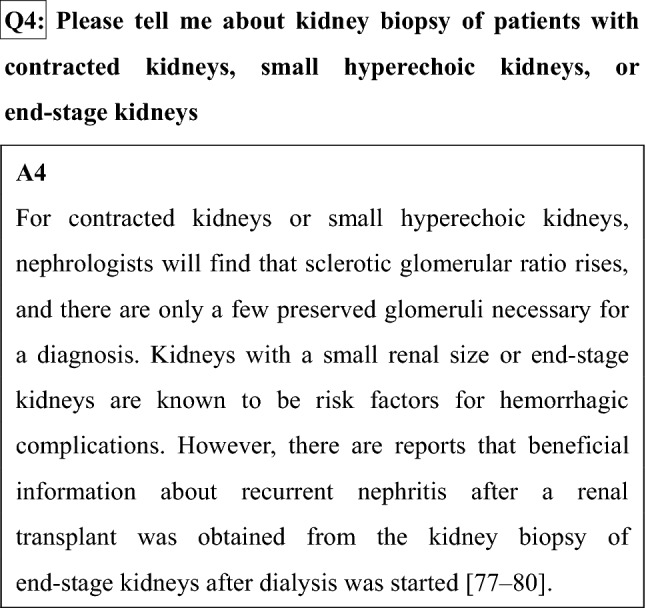



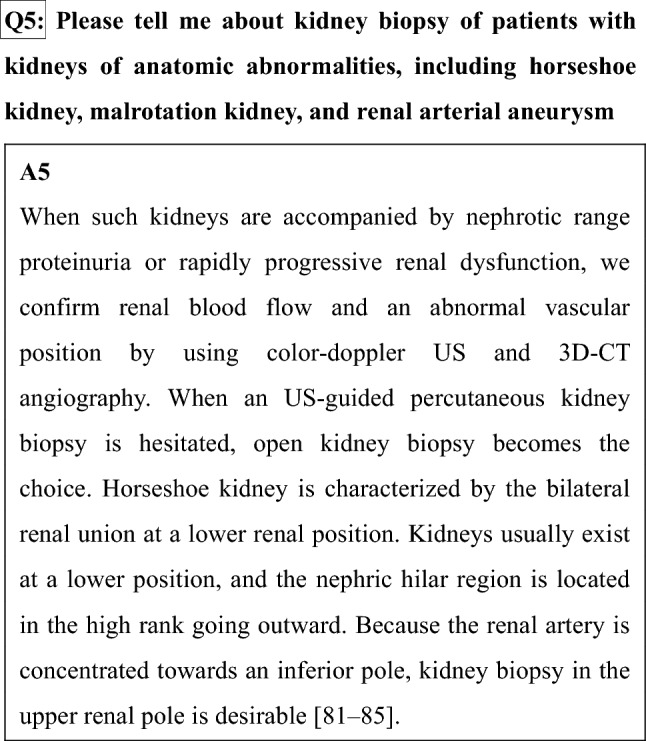



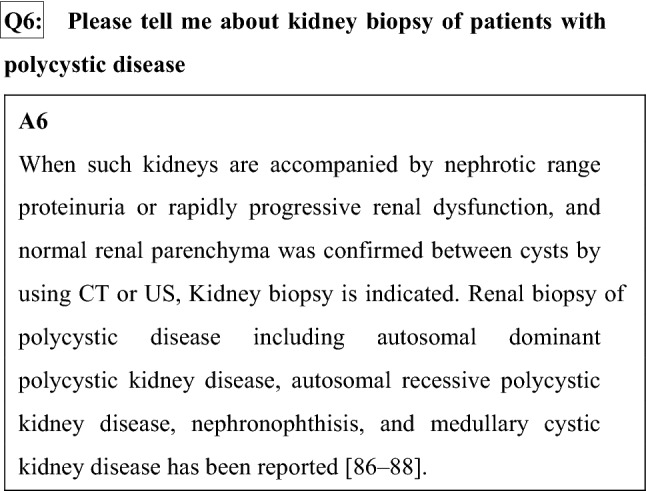



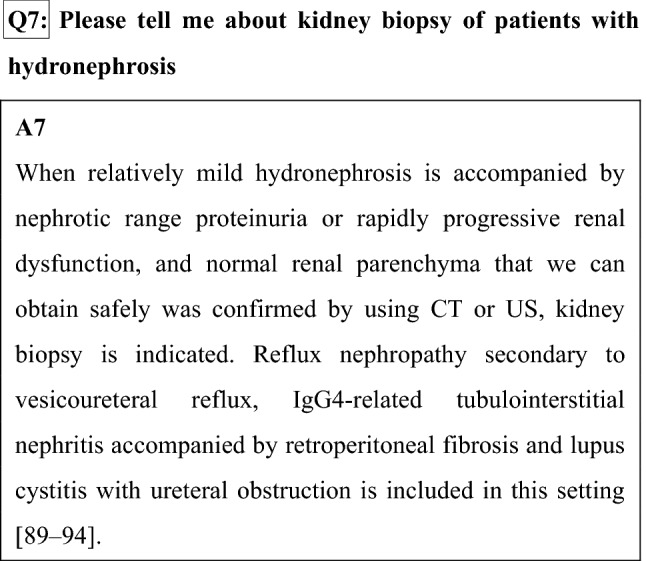



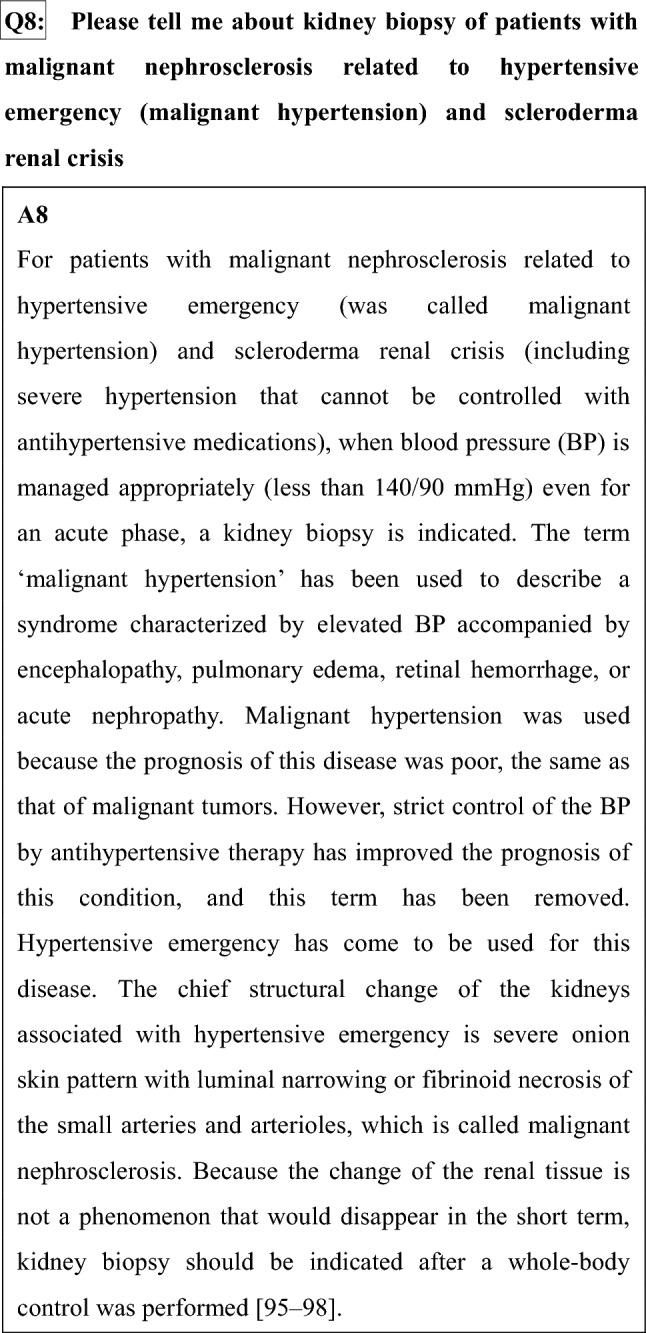



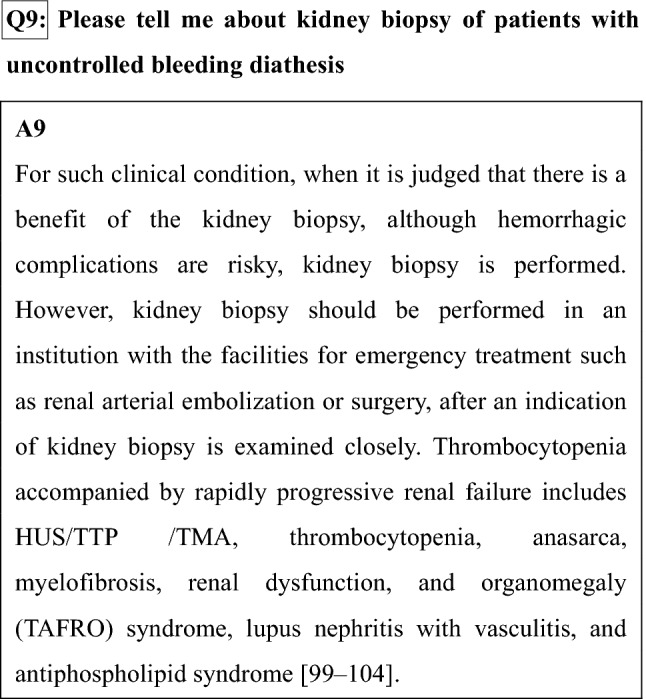



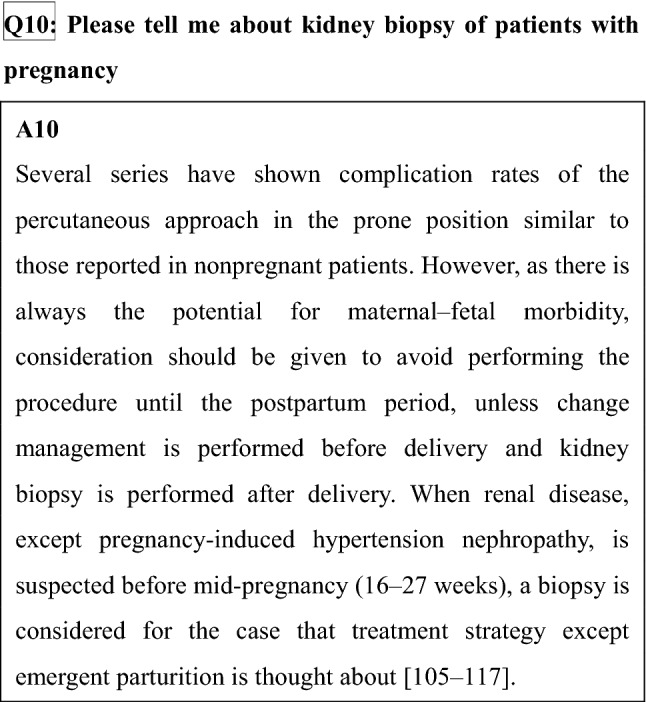



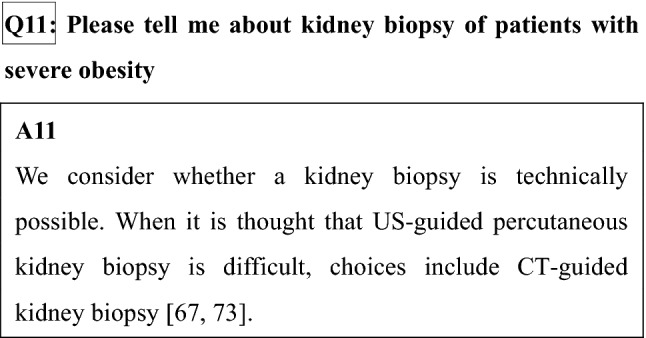



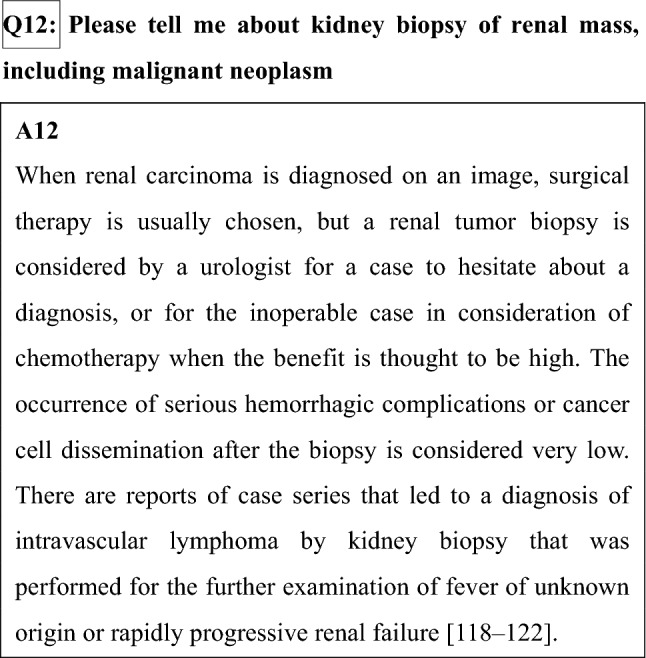



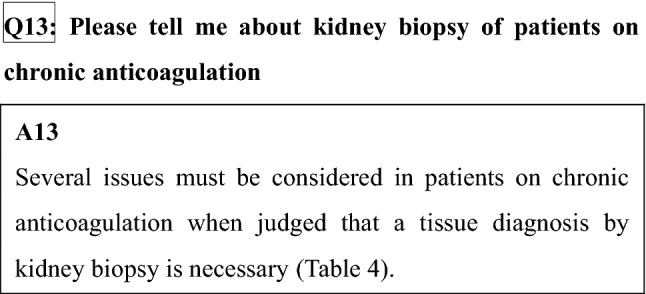

- For patients on chronic anticoagulation, kidney biopsy usually cannot be selected.
- Whether kidney biopsy is essential or necessary for diagnosis, prognosis, and/or management must be discussed in the conference conducted at the institute.
- If anticoagulation is temporarily stopped (e.g., mechanical heart valves), the risk of thrombosis must be judged in consideration of an individual situation, often in consultation with hematology and cardiology.
- If anticoagulation is continued, the risk for bleeding after kidney biopsy must be evaluated in consideration of an individual situation. Kidney biopsy should be performed in an institution with the facilities for emergency treatment [123–125] (Table 4).

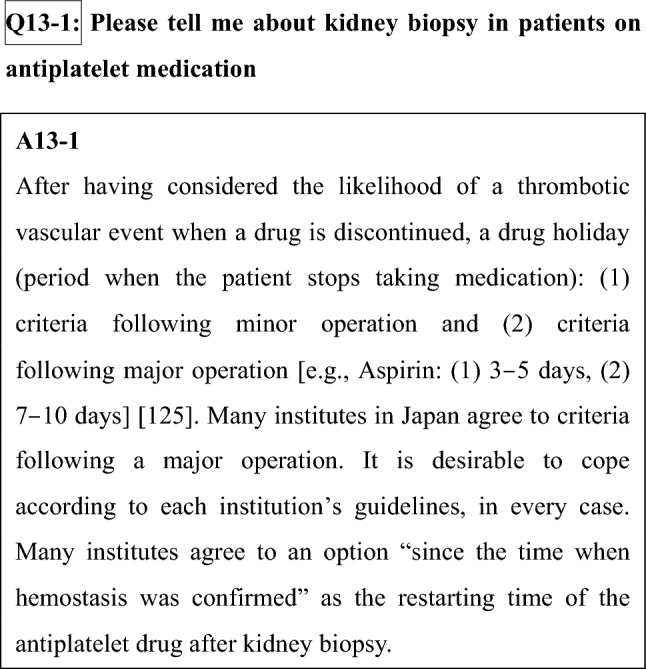



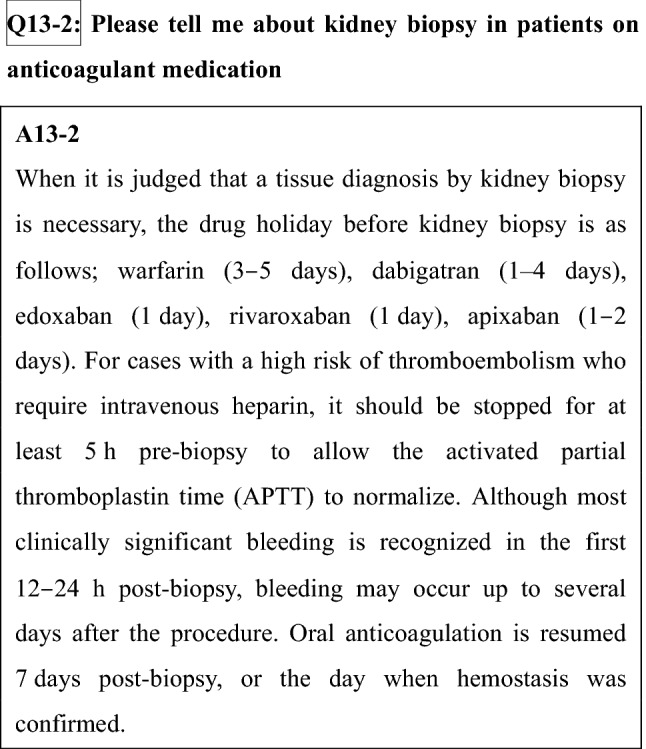



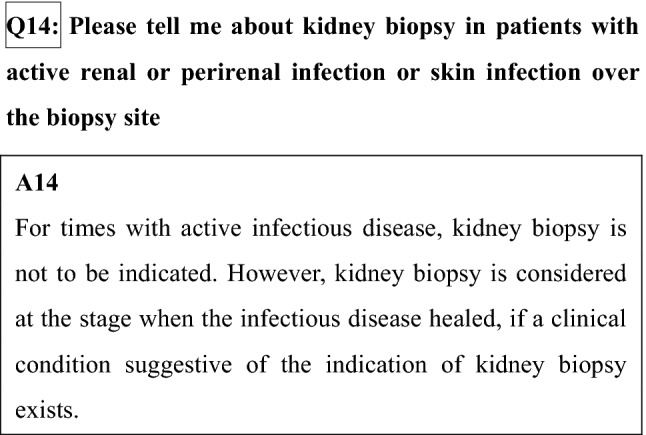



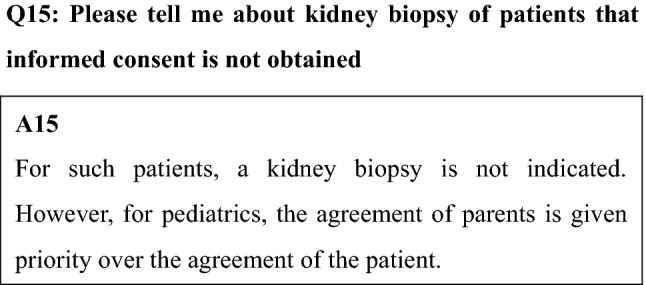



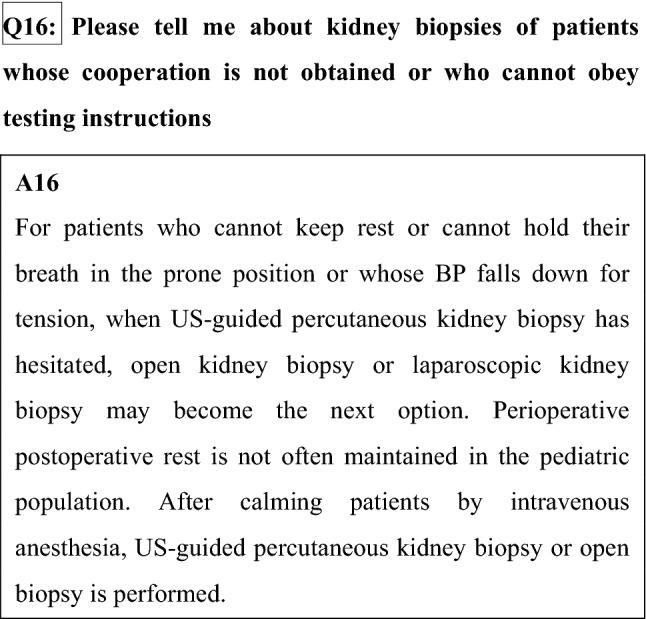


**Table 4:** Chronic anticoagulation and drug holiday before kidney biopsy including two types of options in Japan

Drug	Drug holiday
Antiplatelet medication	
Ticlopidine	① 5–7 days, ② 10–14 days
Clopidogrel	① 5–7 days, ② 14 days
Cilostazol	① 1 day, ② 2–4 days
Icosapentaenoic acid	7–10 days
Beraprost	2–3 days
Sarpogrelate	1–2 days
Aspirin	① 3 days, ② 7–10 days
Dipyridamole	1–2 days
Prasugrel	① 5–7 days, ② 14 days
Anticoagulant medication	
Heparin	1 day
Dalteparin	1 day
Warfarin	3–5 days (intravenous heparin)
Dabigatran	1–4 days
Edoxaban	1 day
Rivaroxaban	1 day
Apixaban	1–2 days (intravenous heparin)
Vasodilator	
Limaprost	1 day
Coronary vasodilator	
Dilazep hydrochloride	1 day

## Chapter 3: Informed consent and explanation document to the patients

### Informed consent in kidney biopsy

#### Overview

Kidney biopsy is a gold standard for renal disease diagnosis and is the testing that we cannot miss in renal disease practice. However, it is invasive testing, and adequate informed consent is necessary. With respect to the nephrologist, it is necessary to explain the possible complications by the testing procedures, including hemorrhagic complications, in addition to the benefits of kidney biopsy to the patients. With respect to the patients, it is important to consent to kidney biopsy based on their own intention after having understood the benefits (merits) and disadvantages (demerits) of kidney biopsy explained by a physician [126].

In Japan, informed consent is obtained before kidney biopsy, and kidney biopsy is performed after, as a general rule, having acquired an agreement by letter. In this issue, the informed consent is commented by a question and answer method.
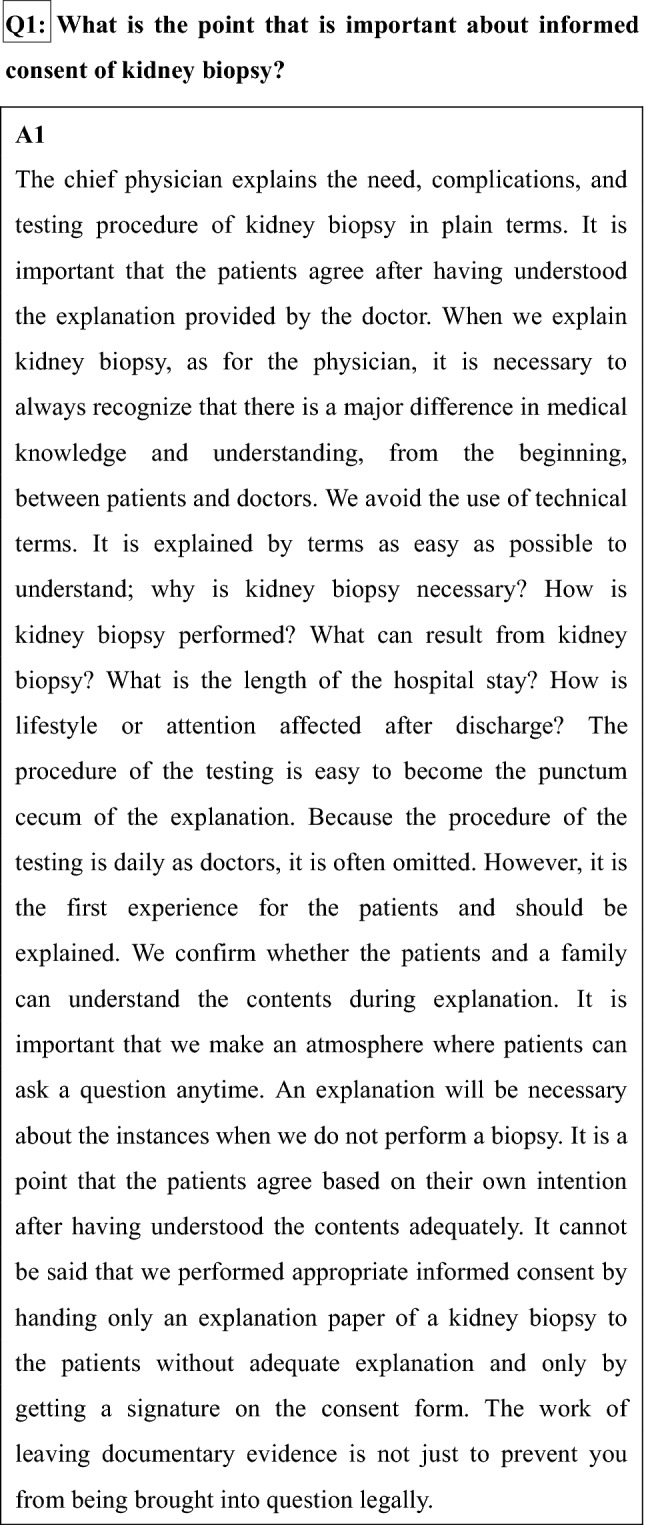



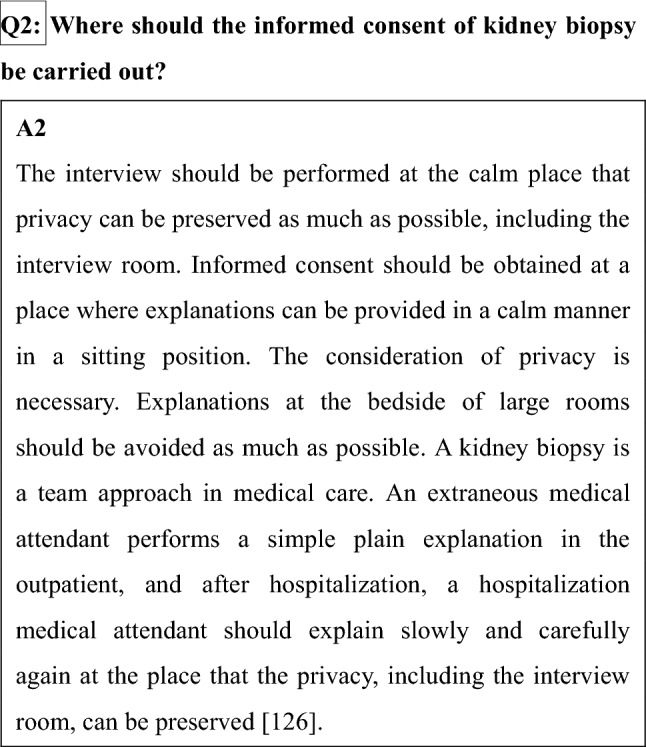



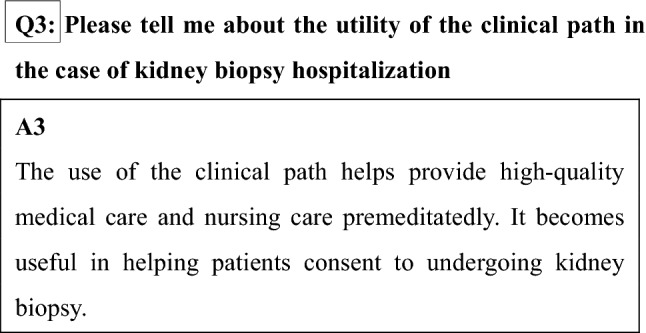



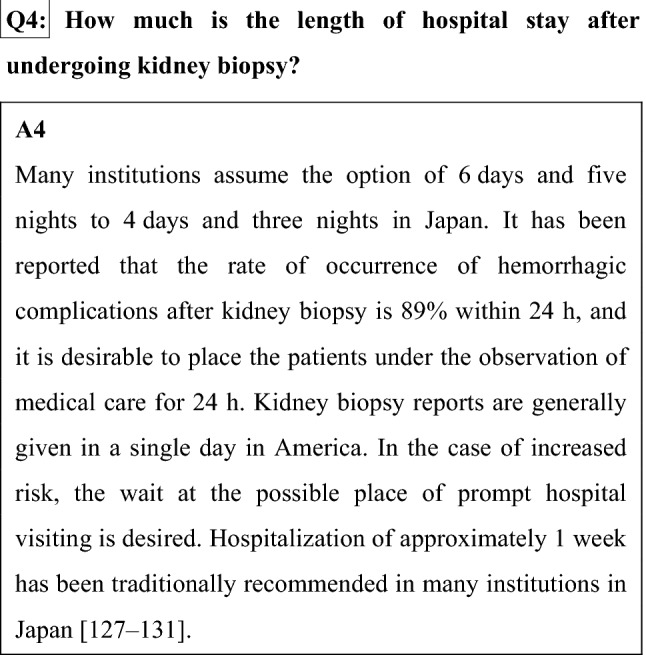



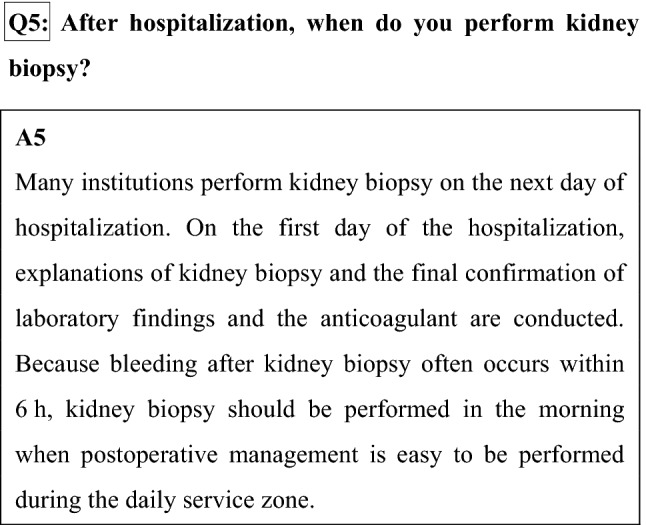



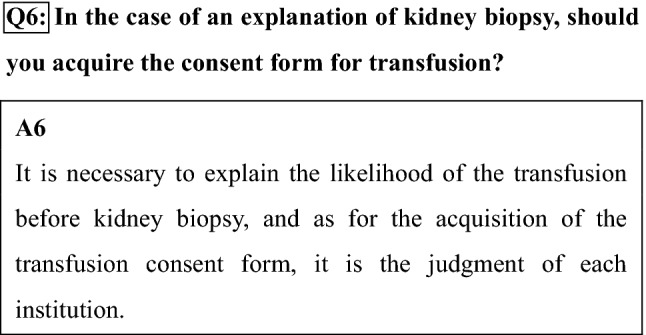


### Explanation document to the patients


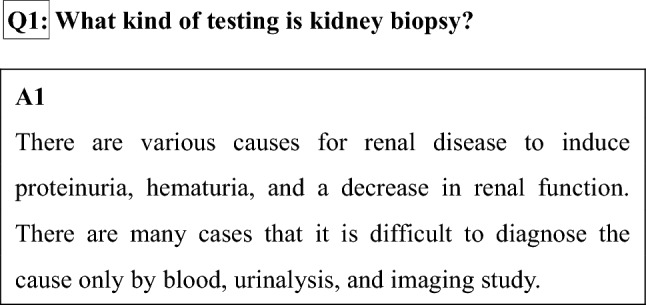


We take some kidney tissue by using the needle with the core size of the ball-point pen, observe it with a microscope, and clarify a cause of renal disease occurring in kidney. If a cause of the illness is understood, we can suggest an optimal therapy. A procedure or an operation to take out kidney tissue is named kidney biopsy.
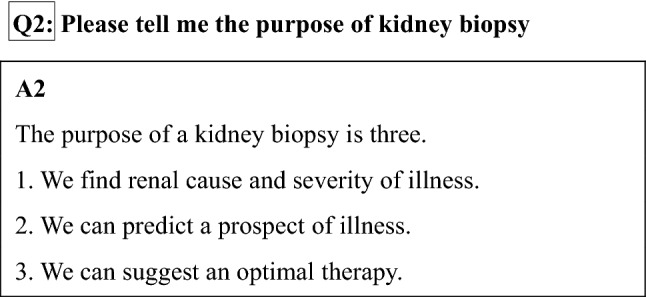

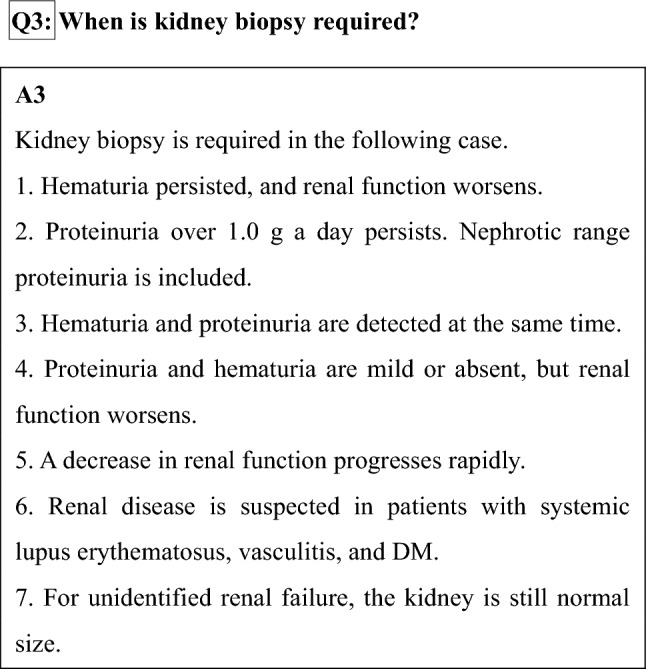



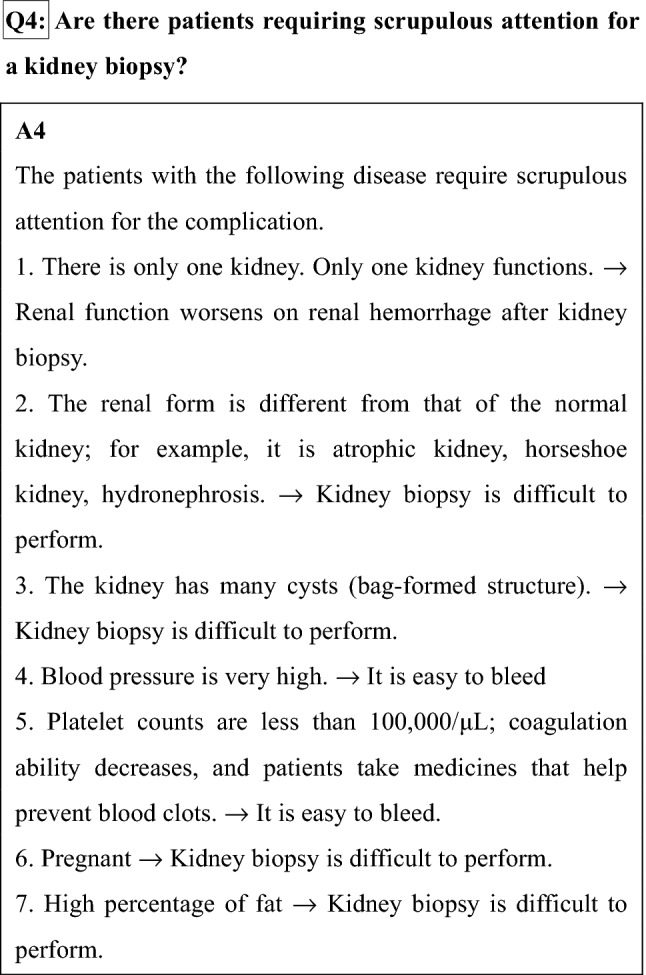



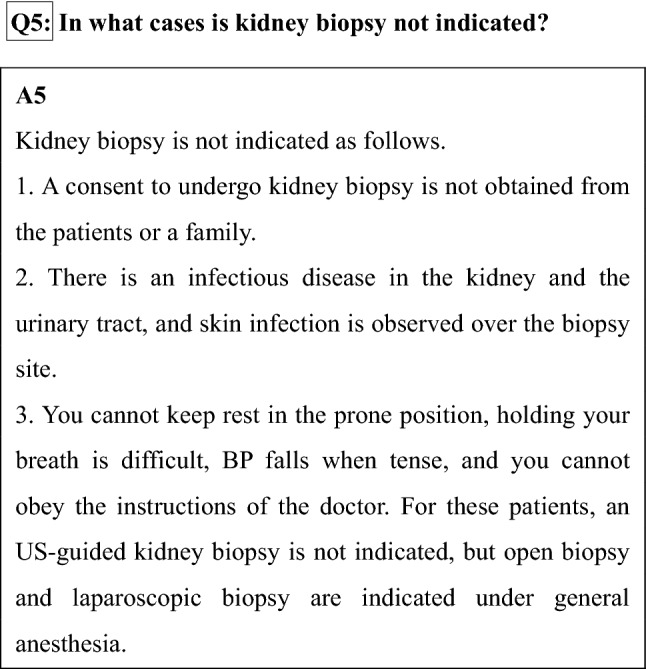



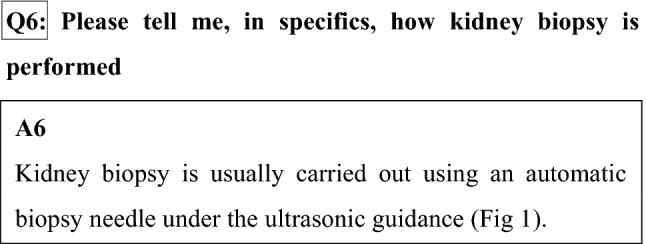

1. We put an indwelling needle for intravenous feeding in the blood vessel of the arm before testing. An antimicrobial agent and/or hemostatic are usually given before testing. When BP falls or you came to feel sick during testing, a drug is given through an indwelling needle.
2. We cancel your diet before the testing. This is because you come to feel sick, and you may vomit by the pressure from a back hemostasis.
3. There is the kidney at the position near a back. You lie on your face and the stomach. A renal place is confirmed by US. From the skin of the back surface to the renal surface, a local anesthetic is injected in place to prick with a needle. We cut about a 2–3 mm opening in the skin surface. This section may remain as a minimal wound subsequently.
4. The thickness of the needle taking the renal tissue is a core size of the ball-point pen, and the length is around 2 cm. When a needle is inserted, there is no pain, but there is the sense that the back is pushed. When the needle reaches the kidney, we signal you. Please hold your breath for 5-10 s. We take the renal tissue at that moment. You hear a clicking sound at the moment that we take the renal tissue. Because there is no pain, do not worry. We conduct this operation 2–4 times.
5. When kidney biopsy is completed, we exert pressure from the back for 10–15 min to stop bleeding.
6. The testing is completed in approximately 30 min. After testing, you turn over on your back. Rest is required in a bed for 6–24 h. Eating and drinking after the testing is performed lying down. Urination and the defecation are carried out on the bed, too. When urination is difficult, we may use a tube called a urethral catheter. After testing, fever may occur. The cause is considered absorption fever occurring when the hematoma that occurred after a biopsy is absorbed.
7. For 4 weeks from the next morning, walking is possible, but please avoid running up the stairs, and please avoid intense, laborious work to avoid exerting stress on the area that was affected by the procedure.
8. With respect to the method of kidney biopsy performed in Japan, an automatic biopsy needle is now used under the ultrasonic guidance in almost all institutions. Kidney biopsy is considerably safer than when performed blindly, and it may be said that it is an established testing method. However, when it may be hard to obtain renal tissue, we may cancel testing on the way without overdoing it. When we cannot obtain renal tissue, or when glomeruli necessary for a diagnosis are not included, we may make a testing plan again.

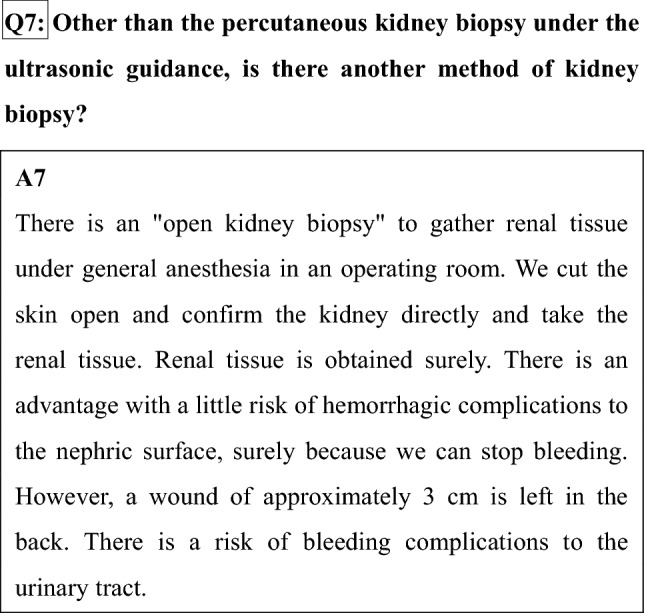


**Fig. 1:**
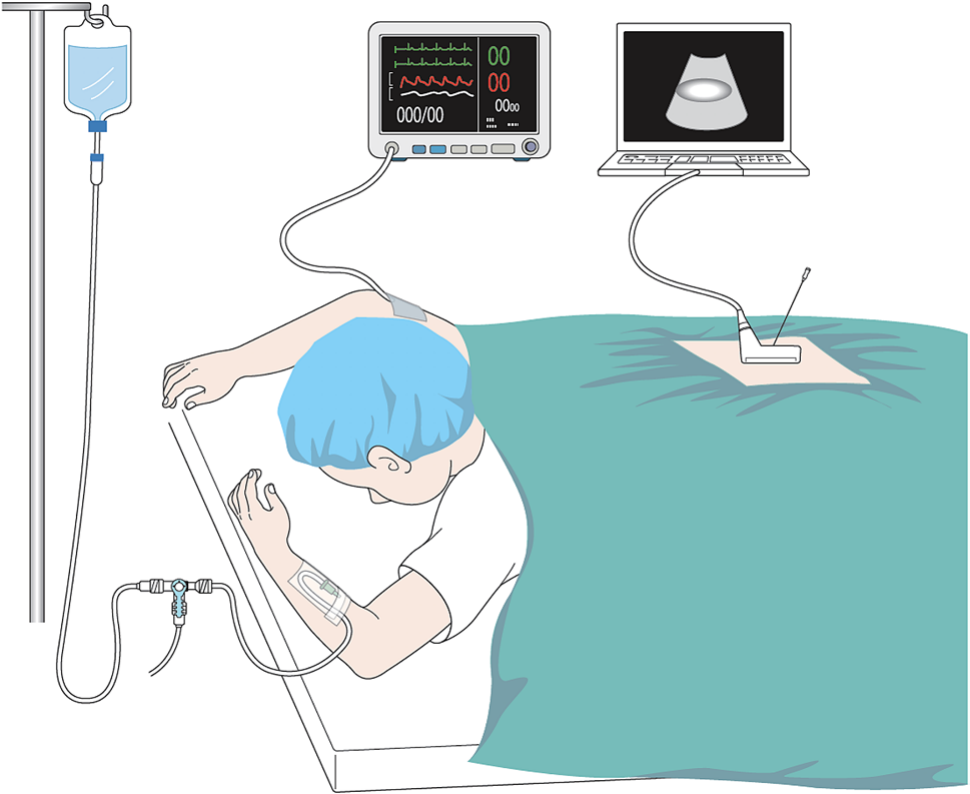
How to do a kidney biopsy

There is a "laparoscopic kidney biopsy," which takes the renal tissue while confirming the kidney using laparoscopy as other methods directly (Fig. 1).

When there is the high-risk clinical condition and hemorrhagic complications by percutaneous kidney biopsy, when renal tissue is not gained by percutaneous kidney biopsy, "opening kidney biopsy" or "laparoscopic kidney biopsy" is chosen.


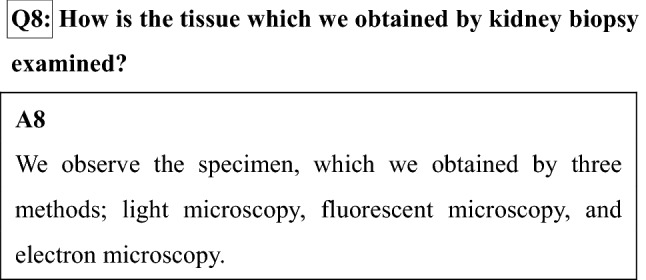


By light microscopy, we can observe the whole, including glomeruli, renal tubules, and the blood vessels, and can obtain basic information.

By fluorescent microscopy, we observe the presence or absence of deposition and a deposition place of immunoglobulin, including IgG, IgA, and IgM, and complements, such as C3 and C1q.

By electron microscopy, we confirm the cellular internal structure, including glomerular and tubular structure, and a deposit causing nephritis, which spreads approximately 15,000 times.

After performing three tests, a diagnosis of renal disease is made.


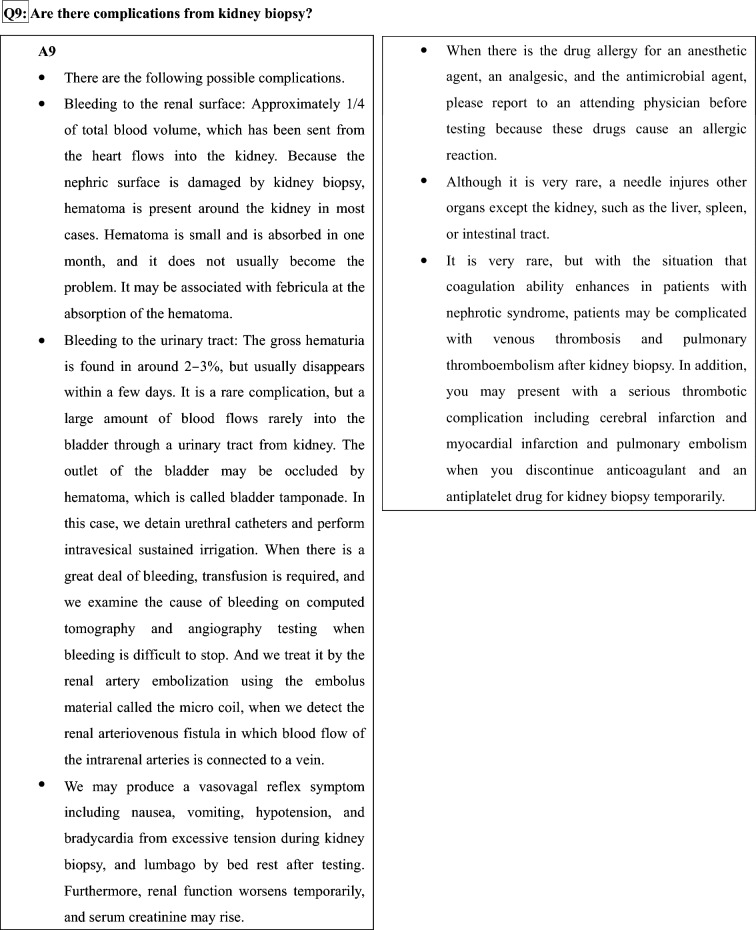


According to questionnaire survey by the Japanese Society of Nephrology for kidney biopsy that was performed in Japan from 2015 through 2017, out of 15,657 adult patients who underwent kidney biopsy by a nephrologist, transfusion was required in 121 cases (0.8%), hemostasis treatment by renal artery embolization in 31 cases (0.2%), gross hematuria with no treatment in 431 cases (2.8%), vesicoclysis in 56 cases (0.4%), death in one (0.006%). Close evaluation of the death cases clarified that bleeding after kidney biopsy is not a direct cause, but the overall status of these cases was poor before kidney biopsy and worsened after kidney biopsy.

## Chapter 4: Pre-biopsy evaluation


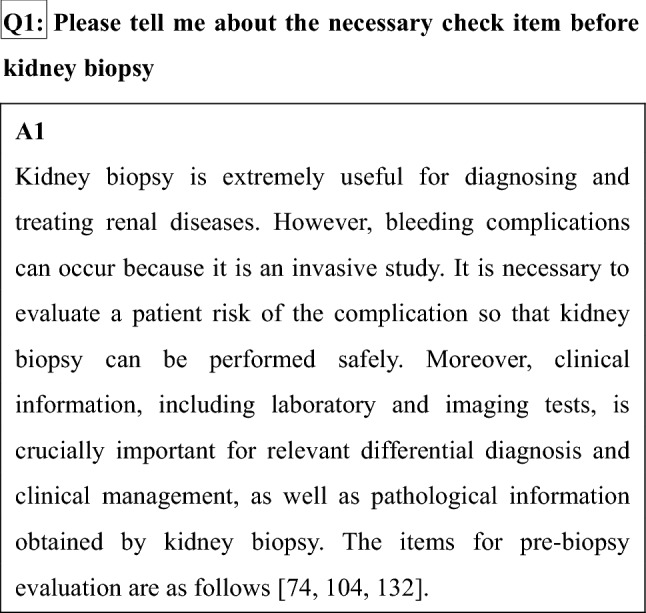

1. Medical history
  ① Detailed history of present illness.
  ② Family history of renal diseases.
  ③ Past medical and social history.
  ④ History of patient medication.
2. Physical examination
3. Blood test

① Complete blood cell count
  Erythrocyte transfusion is considered for severe anemia before kidney biopsy. The cutoff value of Hb is 7–8 g/dL. Platelet transfusion is considered for severe thrombocytopenia with platelet count less than 50,000/μL.
② Coagulation study
  Tests for prothrombin time (PT), APTT, fibrinogen, and fibrin/fibrinogen degradation products (FDP) (or D-dimer) are recommended for pre-operative screening. When a coagulation abnormality is found, close examination and adequate treatment are required before kidney biopsy. When a thrombotic tendency is pointed out, especially in high-risk patients with nephrotic syndrome, screening tests for deep vein thrombosis and pulmonary embolism are also considered.
③ Biochemistry
  Serum tests include total protein, albumin, urea nitrogen, creatinine, uric acid, AST, ALT, LDH, and electrolytes (Na, K, Cl, Ca, P, and Mg). Estimated GFR by using serum creatinine or cysteine C values are important to evaluate renal function. Arterial blood gas analysis (including anion gap) is also helpful for the differential diagnosis of kidney diseases with acid–base abnormality.
④ Blood sugar (glucose) test
  As well as fasting plasma glucose (sugar), HbA1C and glycoalbumin are useful for evaluation of hyperglycemic conditions.
⑤ Immunology
  Immunological tests include immunoglobulin (IgG, IgA, IgM, IgG4), complement (CH50, C3, C4), autoantibody (antinuclear antibody, ds-DNA, SM, RNP, ANCA, GBM, anticardiolipin, lupus anticoagulant), serum monoclonal protein.
⑥ Endocrinology
  Endocrinological examinations include renin, aldosterone, and BNP.
⑦ Tests for infection
  HBV, HCV, syphilis (RPR/TPHA), and HIV are screened.
4. Urinalysis
  ① Urinary qualitative test (dipstick test).
  ② Urinary sediment.
    Dysmorphic erythrocytes suggest hematuria with glomerular diseases.
  ③ Urinary quantitative test.
    Urinary protein is measured by using spot urine or 24-h collected urine. NAG, β2MG, and α1MG are useful markers for tubular dysfunction. Selectivity index (SI) is also helpful in the differential diagnosis of nephrotic proteinuria.
5. Imaging test
  Diagnostic imaging includes US, CT, and MRI. Radioisotope examinations are also useful for understanding renal pathophysiology. 99mTc-MAG3, an isotope secreting from proximal tubules, is utilized for evaluating effective renal plasma flow (ERPF) of right and left kidneys. 99mTc-DTPA, an isotope filtrating from glomeruli, is used for the measurement of glomerular filtration rate (GFR) of right and left kidneys.

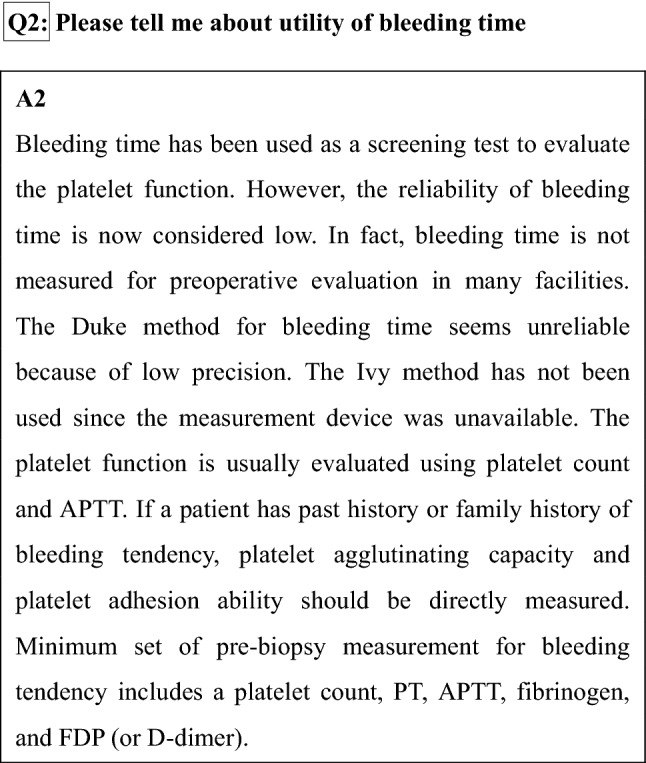


## Chapter 5. Method of kidney biopsy (technique)


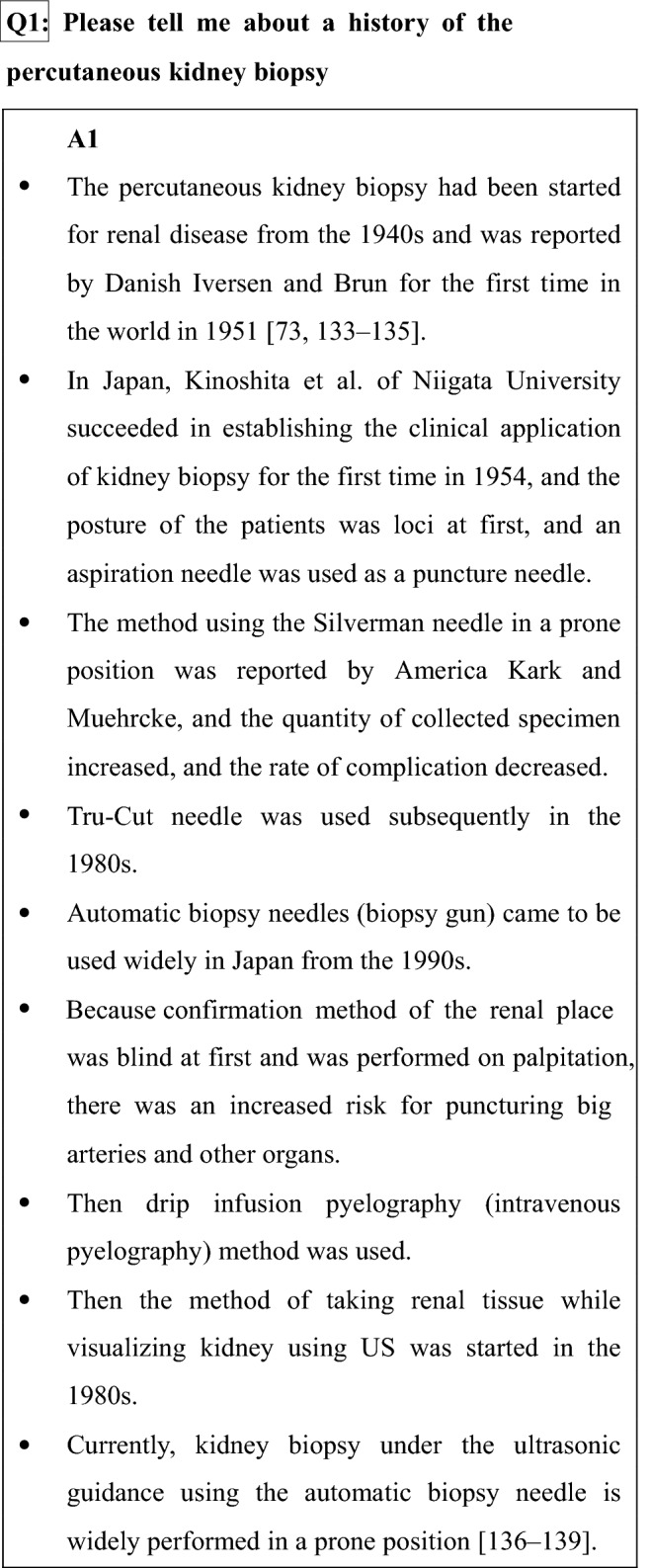



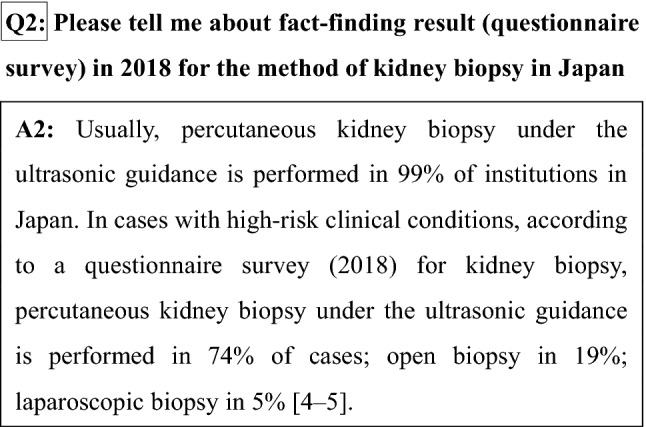



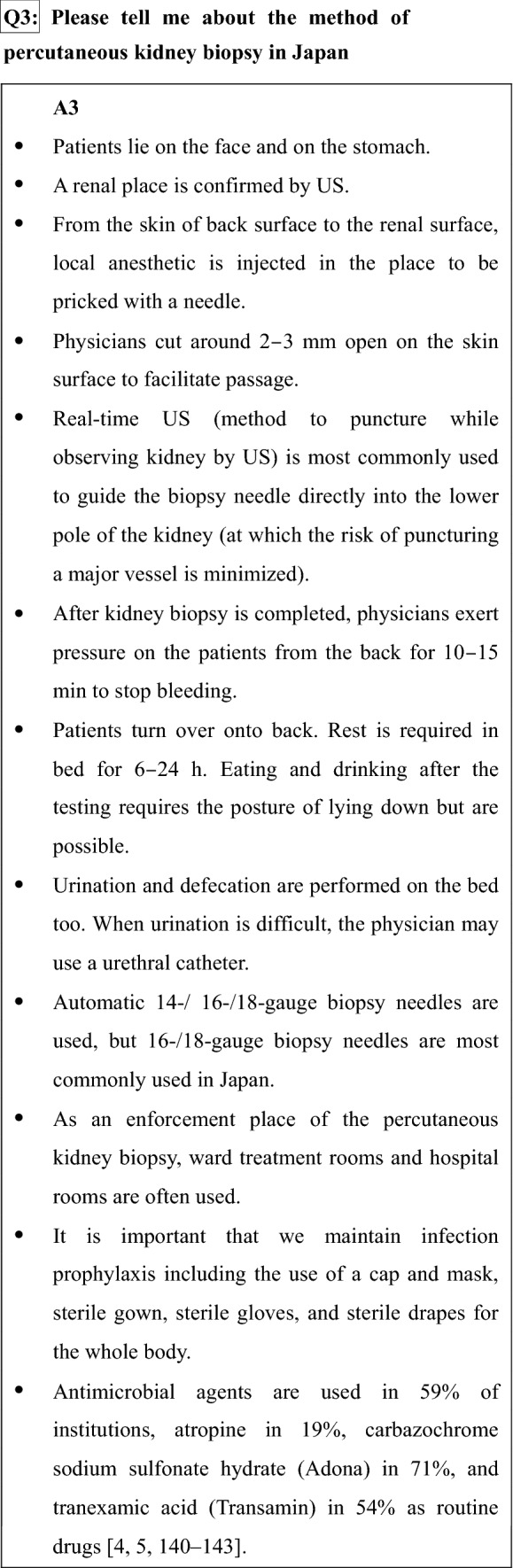



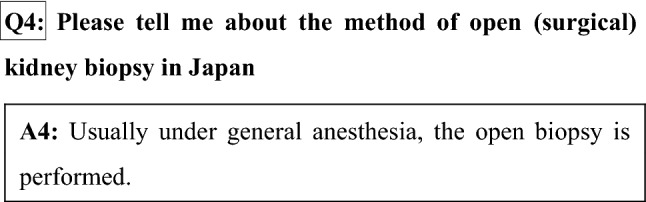

- Setting the patient in lateral jack-knife position, through 3 cm of horizontal incision from 12 rib tip the muscles are divided in each layer to reach the inferior pole of the kidney covered by adipose tissue. Confirming not to damage the peritoneum, the circumrenal fat and Gerota fascia are cut to reach the surface of kidney. The biopsy gun for needle biopsy on the kidney or the wedge incision for block type specimen is used to take a piece of the kidney. After biopsy, hemostasis is securely performed by pressure with the forefinger for 10–15 min. The muscles and skin are closed in layers to finish the procedure. [144, 145].

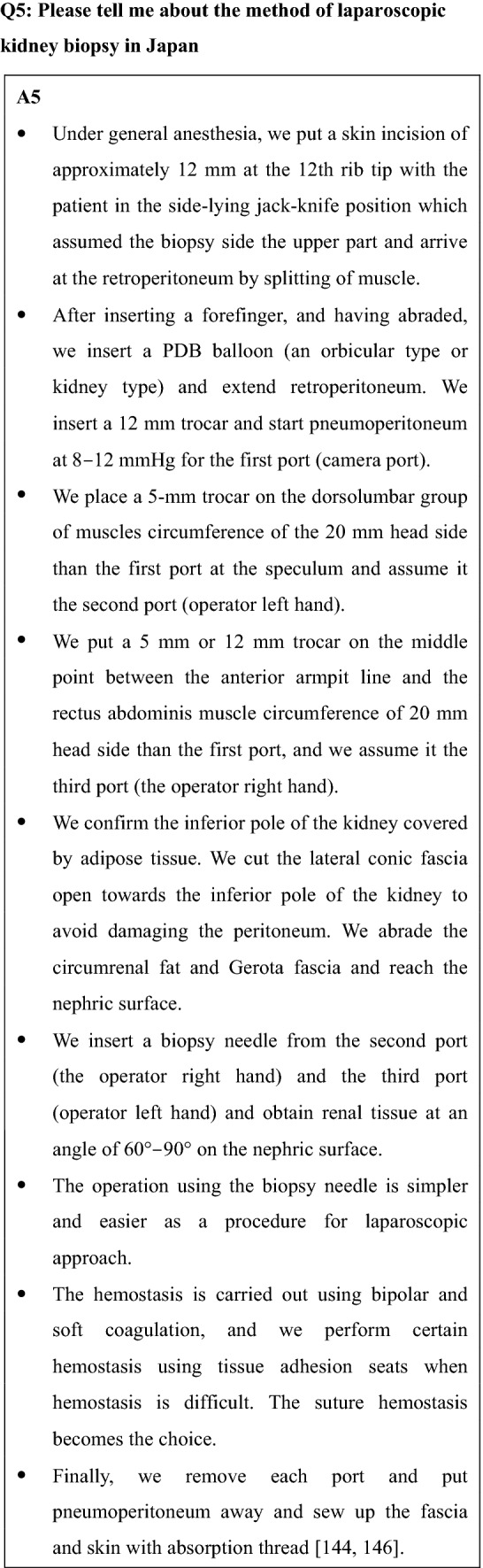



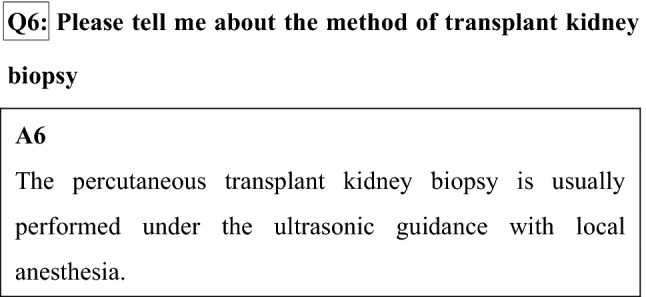

- Because hemostasis pressure can be provided surely as compared with a native kidney biopsy, it is not necessary to discontinue the anticoagulant therapy. However, it is desirable to conduct an examination for coagulation system in advance.
- Under local anesthesia the biopsy needle is put into the kidney to take a piece of the kidney. This may be performed 2–3 times to obtain an adequate specimen.
- Just after the procedure, the physician presses the puncture area for 10–15 min for hemostasis. After that A 1 kg sandbag is put on the puncture area to maintain pressure for an hour. A small pillow is fixed with elastic tape on the area. Thereafter the patient must lie in bed for 6 h or until seen by the doctor. The patient must pay attention for blood in their urine after the biopsy.
- The fixing elastic tape will be removed on next morning. Before discharge a blood count, biochemistry test, and urinalysis are examined. The discharge is permitted after having confirmed that there is no hematoma and hydronephrosis around the renal graft by US [147–149].

## Chapter 6: After care of the biopsy and post procedure observation

Aftercare of the biopsy and postprocedure observation are essential to prevent hemorrhagic complications. After biopsy, bed rest for 6–8 h in an extraneous dressing room is mandated in Europe and America. In Japan, kidney biopsy is performed during hospitalization. Just after the biopsy is performed, pressure is exerted on the back by using both hands and a sandbag for hemostasis. Subsequently, bed rest in the dorsal (supine) position is common [98, 150–160].
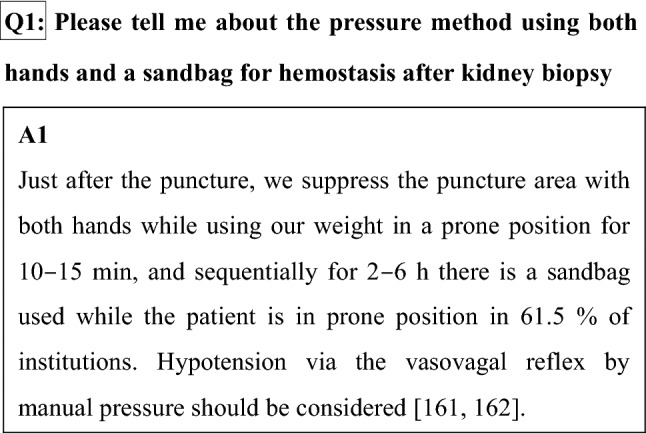

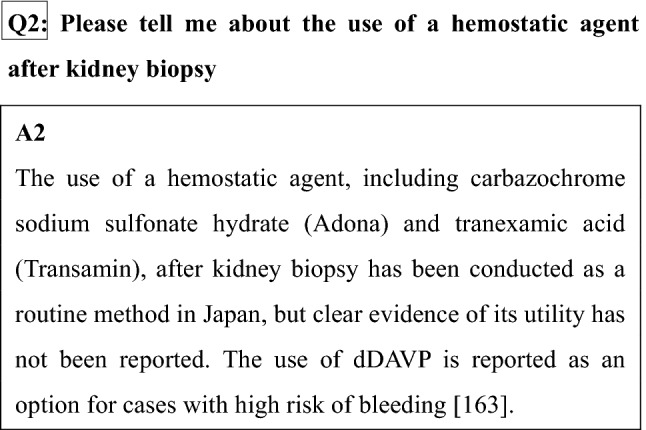

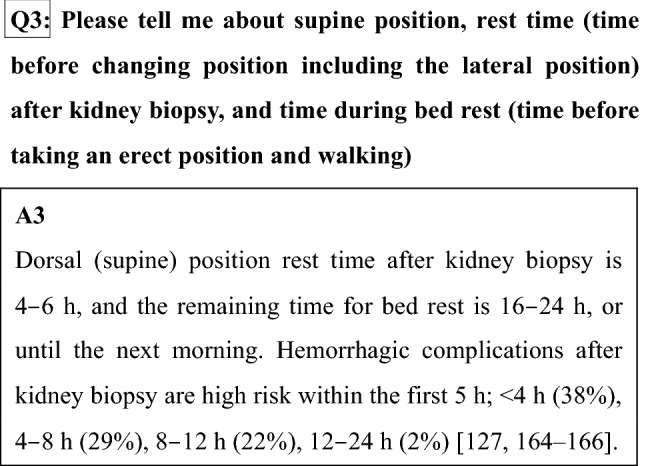



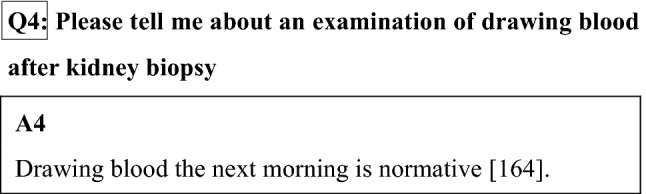



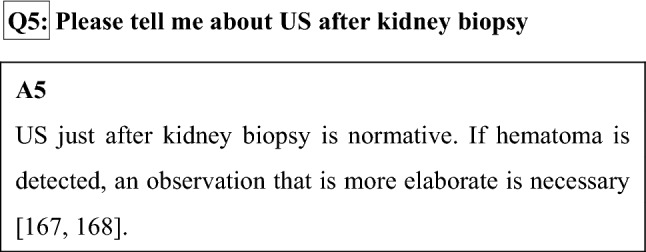



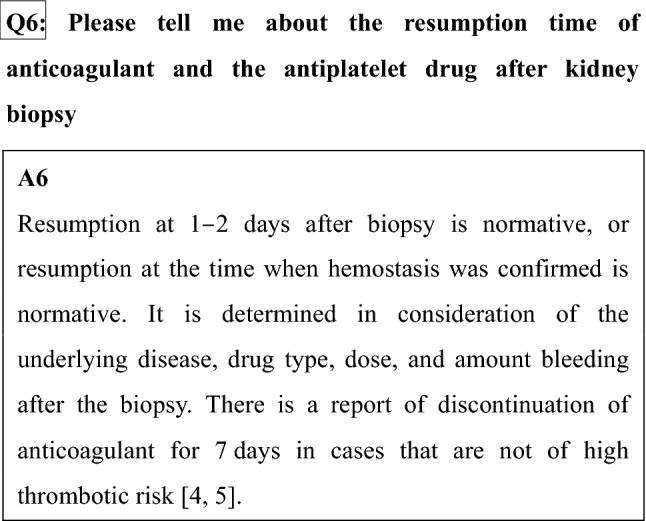



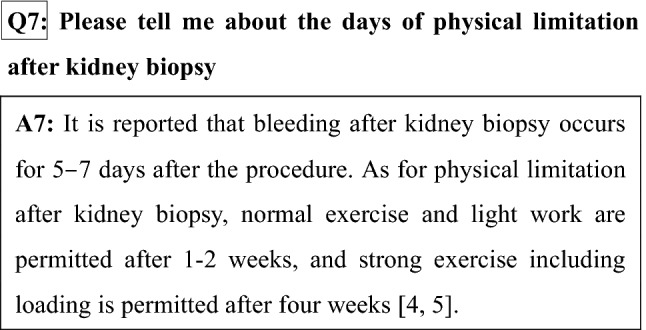



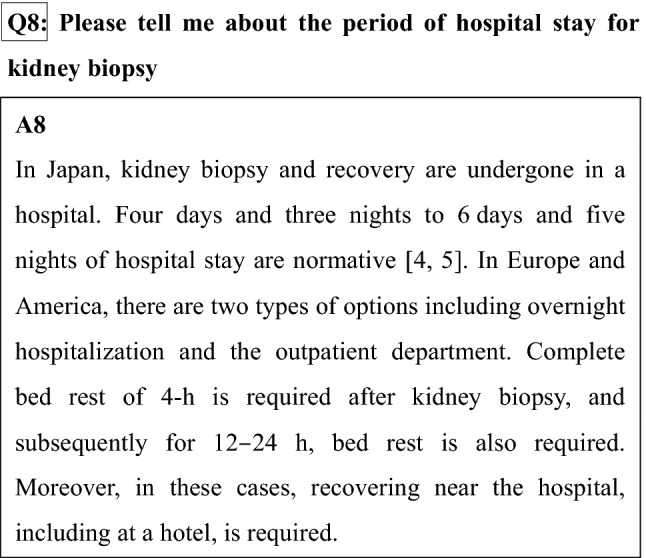


## Chapter 7: Complications

According to the questionnaire survey results that were performed for the publication of this book, among 21,648 kidney biopsy cases that were performed in Japan, gross hematuria after kidney biopsy was found in 511 patients (2.4%), bladder wash was in 79 cases (0.36%), red blood cell transfusion was in 161 cases (0.74%), renal arterial embolization was in 44 cases (0.22%), and death occurred in one case (0.005%). The underlying cause of death in this case was not due to bleeding after kidney biopsy, but the overall status of this case was confirmed poor before kidney biopsy and worsened after kidney biopsy (Table 5) [1, 4, 5, 66, 74, 156, 169–174]. 
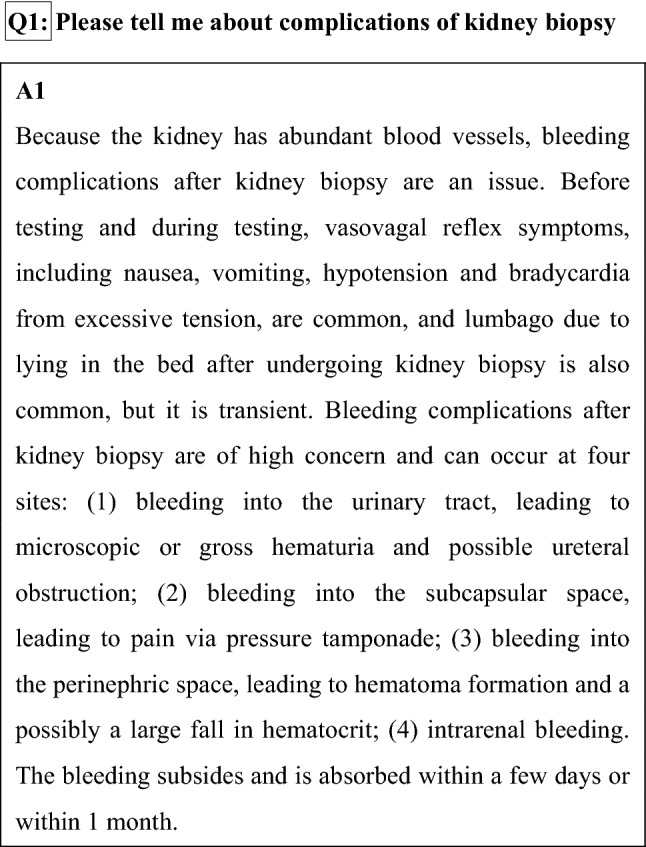



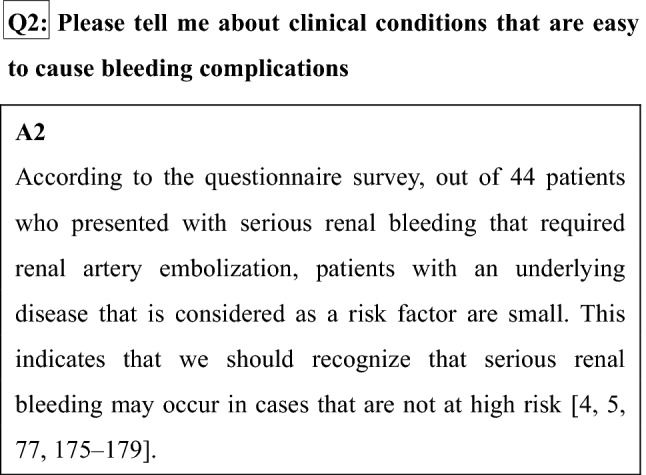



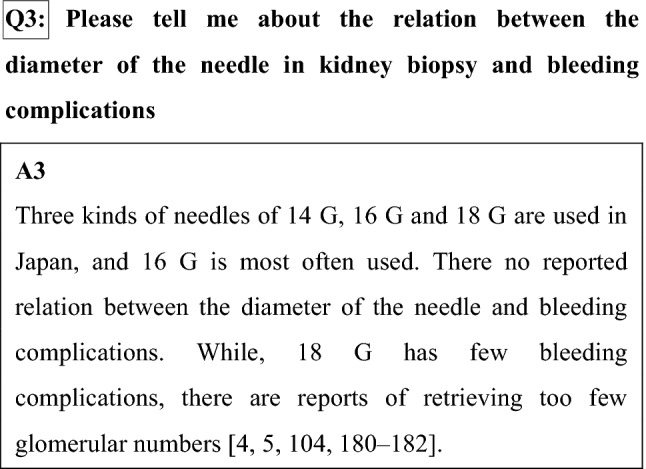


**Table 5:** Bleeding complications after kidney biopsy

	Percutaneous native kidney biopsy	Percutaneous native kidney biopsy	Open biopsy	Transplanted kidney biopsy
	Adult	Children		
Total number of biopsies	15,657	1685	1156	3808
Macroscopic hematuria with no treatment	431 (2.8%)	105 (6.2%)	9 (0.78%)	12 (0.31%)
Erythrocyte transfusion	121 (0.8%)	0 (0%)	4 (0.35%)	2 (0.05%)
Transcatheter arterial embolization	31 (0.2%)	1 (0.06%)	2 (0.17%)	4 (0.1%)
Bladder lavage	56 (0.4%)	9 (0.5%)	0 (0%)	0 (0%)
Nephrectomy	0 (0%)	0 (0%)	0	1 (0.03%)

Results from questionnaire survey for kidney biopsy that was performed in Japan from 2015 through 2017


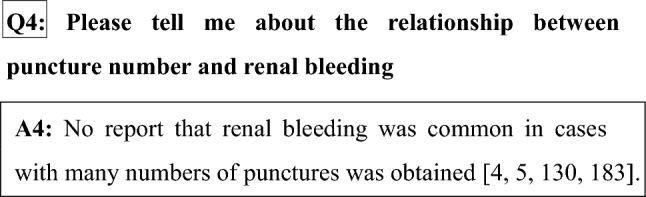



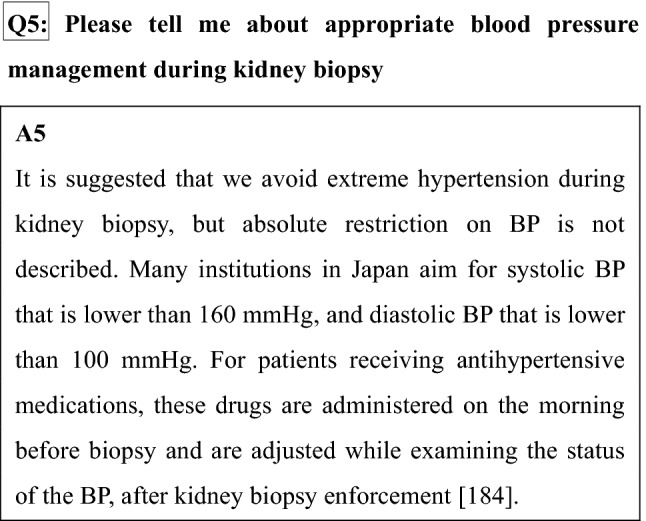



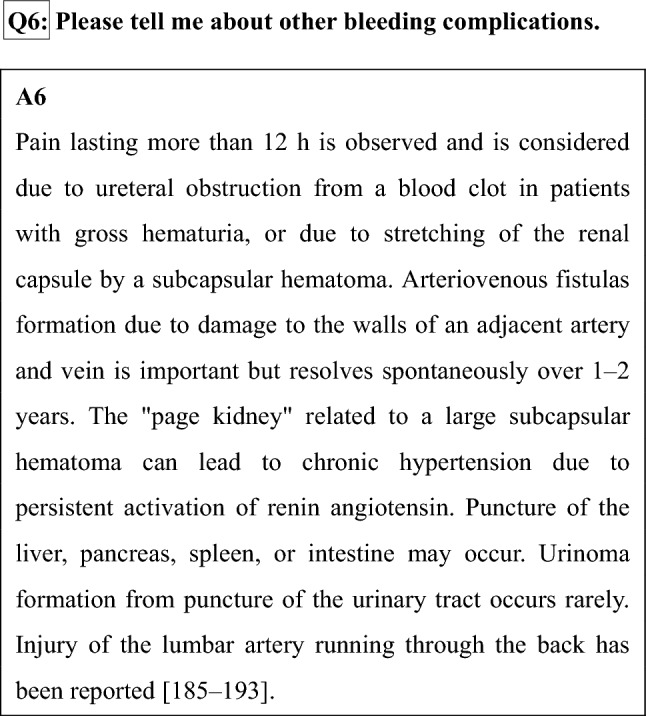



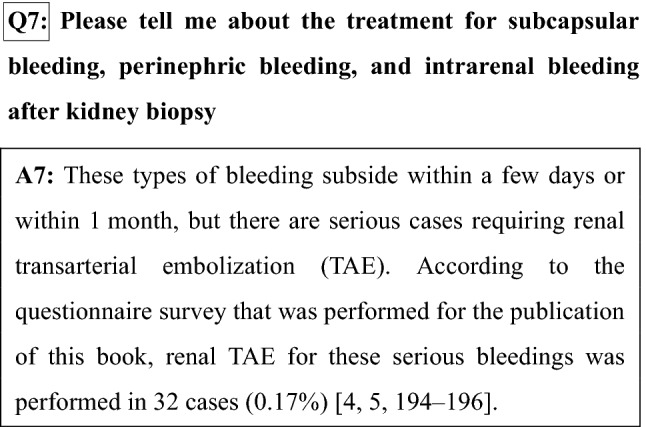



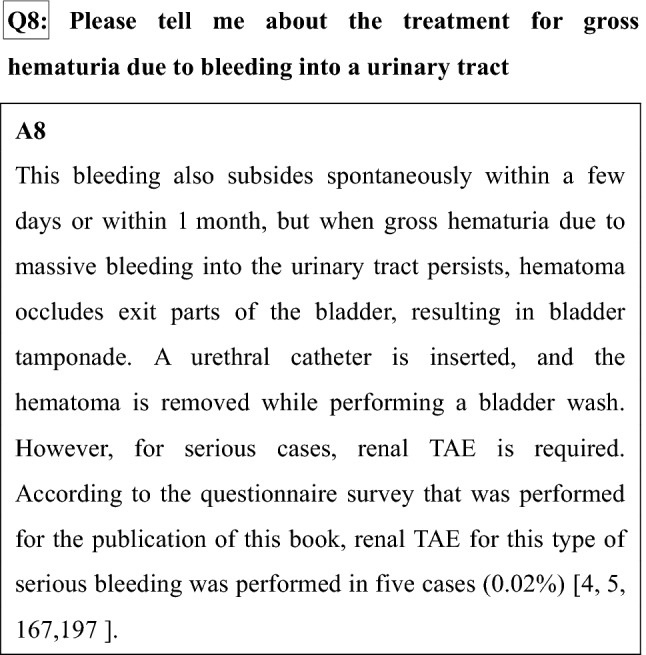


## Chapter 8: Histological evaluation of kidney biopsy specimen

**Fig. 2:**
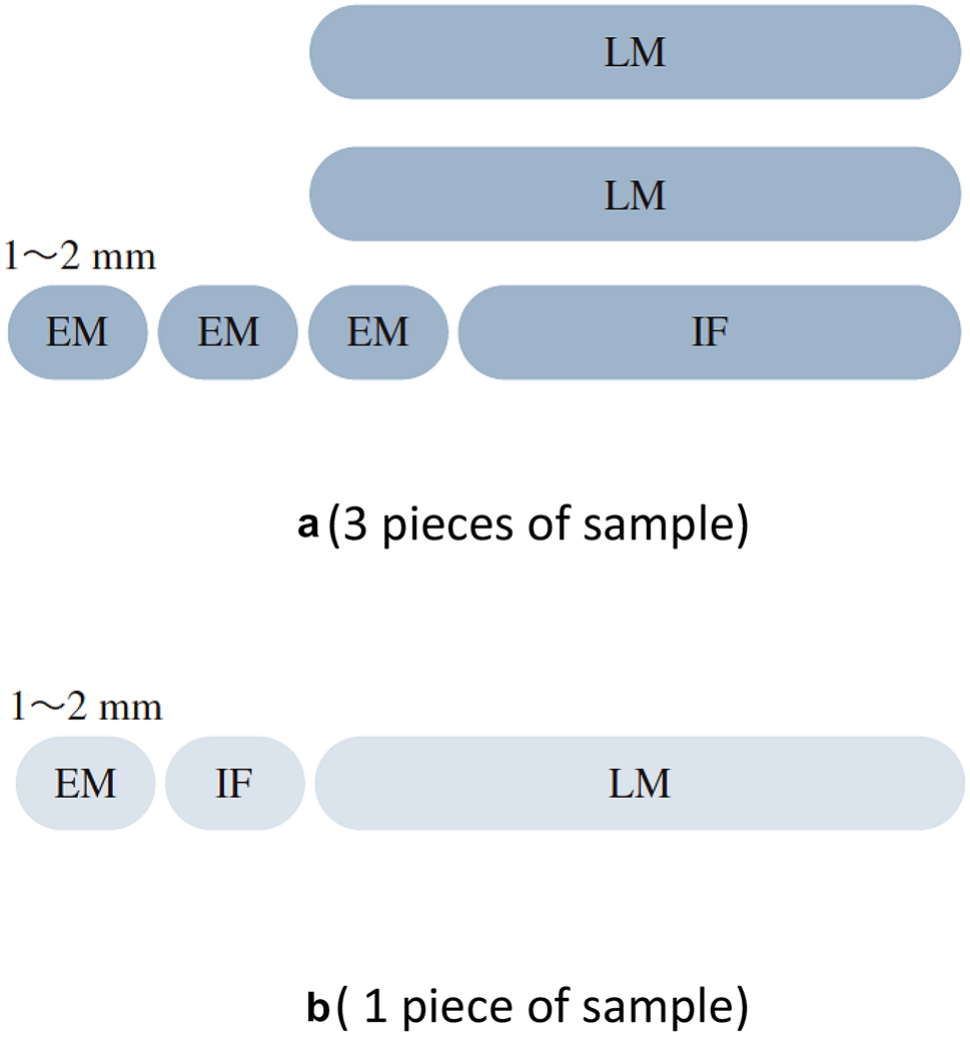
How to divide a sample of the kidney biopsy


Kidney biopsy remains the gold standard to diagnose renal disease and evaluate acute and chronic renal damages. Specimens are processed for the diagnostic approach of light microscopy (LM), immunostaining by immunofluorescence (IF) or immunohistochemistry, and electron microscopy (EM). To minimize the bleeding risk, less passes to obtain tissue is desirable; on the other hand, sufficient quantity of tissue is required for definite diagnosis. When small sample size of renal tissues was obtained, dividing samples appropriately into LM, IF, and EM studies should be carefully considered (Fig. 2). 
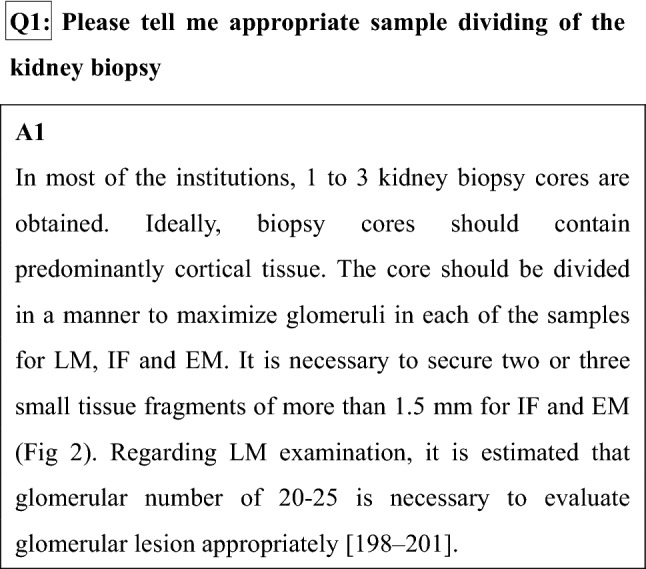



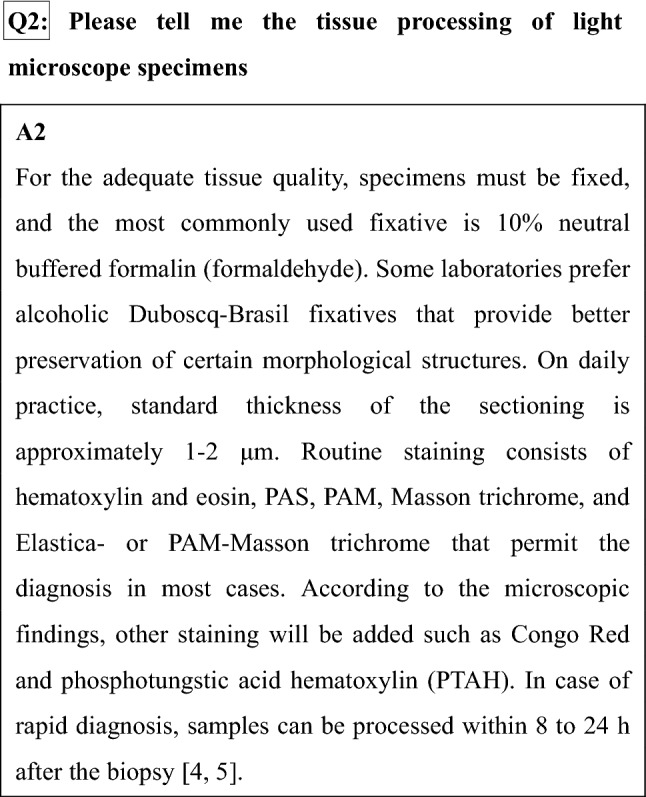



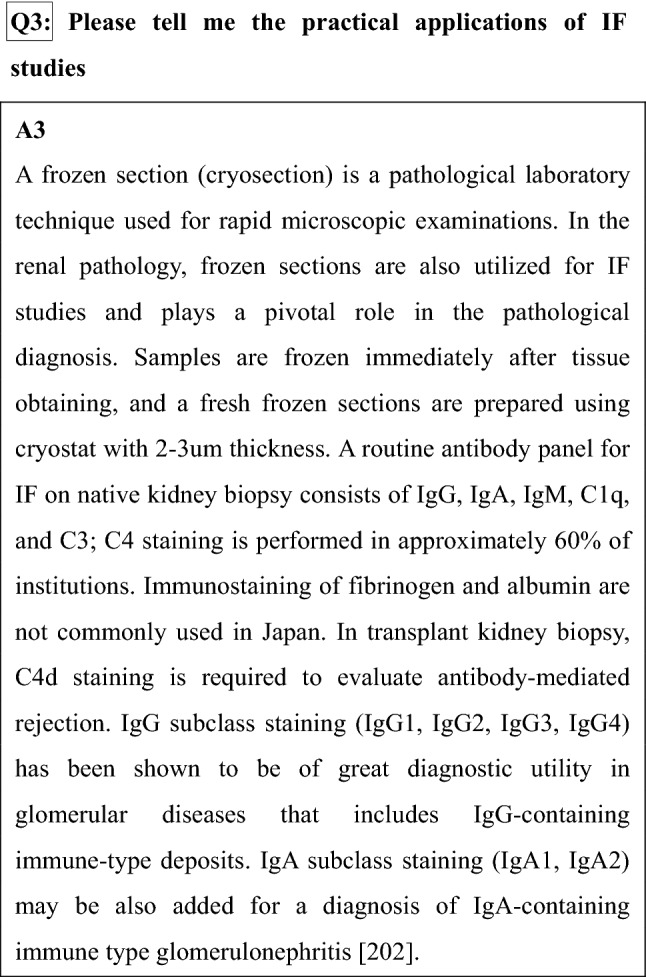



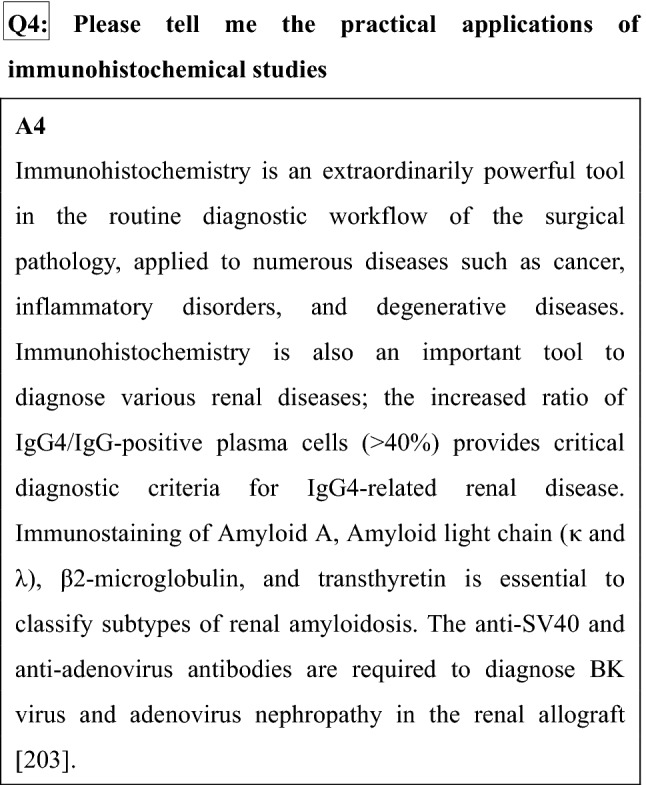


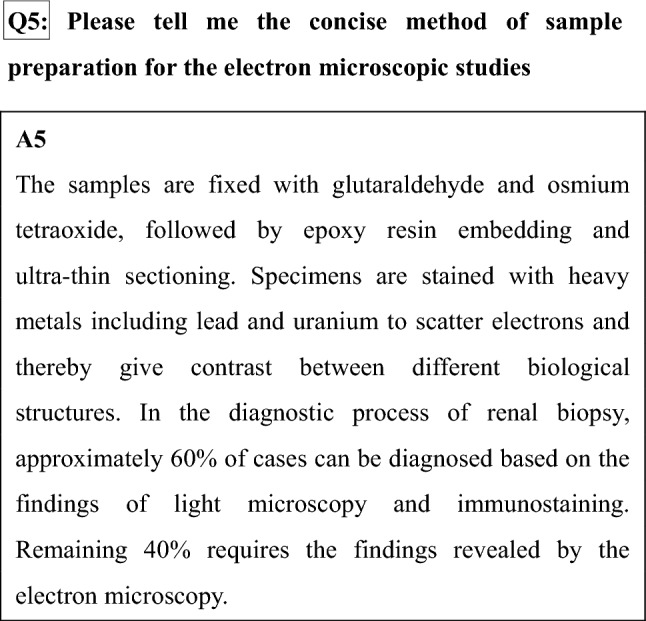



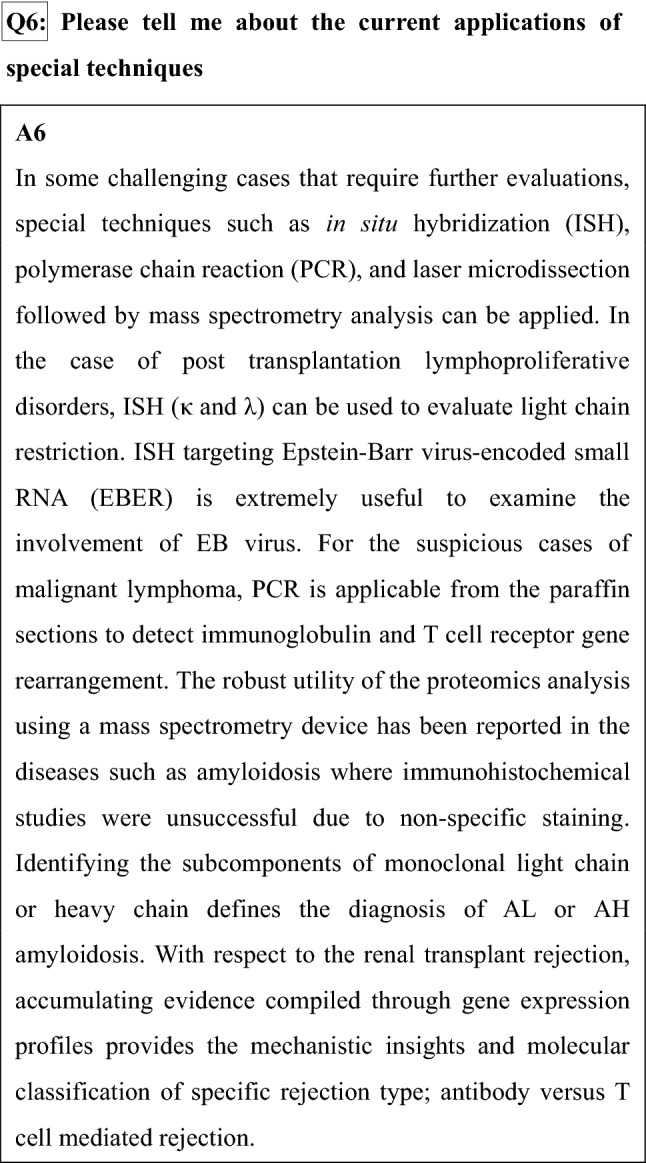



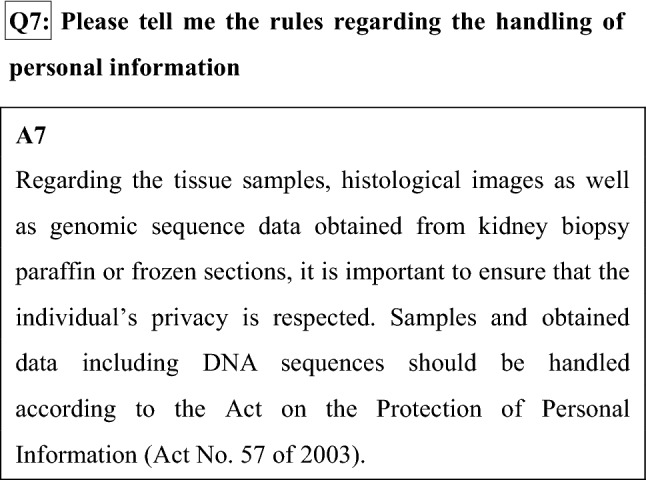


## Chapter 9: Kidney biopsy in children

Kidney biopsy in the pediatric population was reported for the first time in 1958 and has a history of more than 60 years [204]. The procedure has become relatively safe in children as well as in adults owing to technical advances and improvement of medical devices. However, the indication for kidney biopsy must be carefully determined based on benefits and potential risks for serious bleeding complications.

### Indication of kidney biopsy (Table 6)

**Table 6:** Indication of kidney biopsy in children

1. Abnormal urinalysis	1. Isolated proteinuria 0.5 g/gCr or more
	2. Proteinuria and glomerular hematuria
2. Nephrotic syndrome	1. Steroid-resistant nephrotic syndrome
	2. Coexistence of hematuria, hypertension, renal dysfunction, and hypocomplementemia
	3. Congenital nephrotic syndrome
3. Systemic disease with a urinalysis abnormality	1. Systemic lupus erythematosus
	2. IgA vasculitis (Purpura nephritis)
	3. Microscopic polyangiitis
	4. Others
4. Intrinsic acute kidney injury	
5. Others	1. Drug-related disease
	2. Transplanted kidney


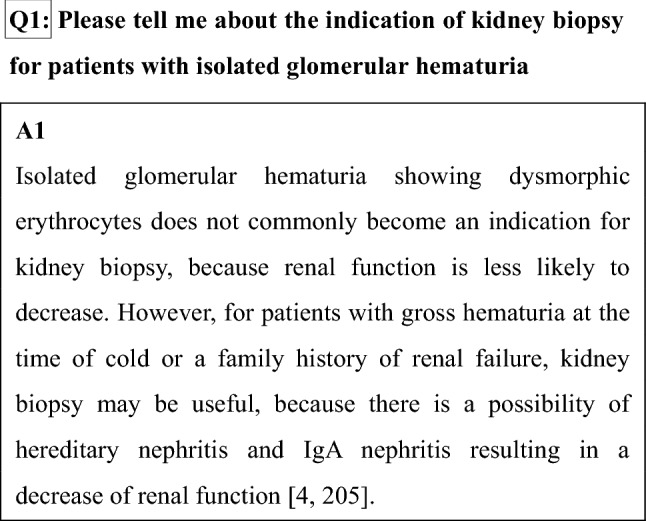



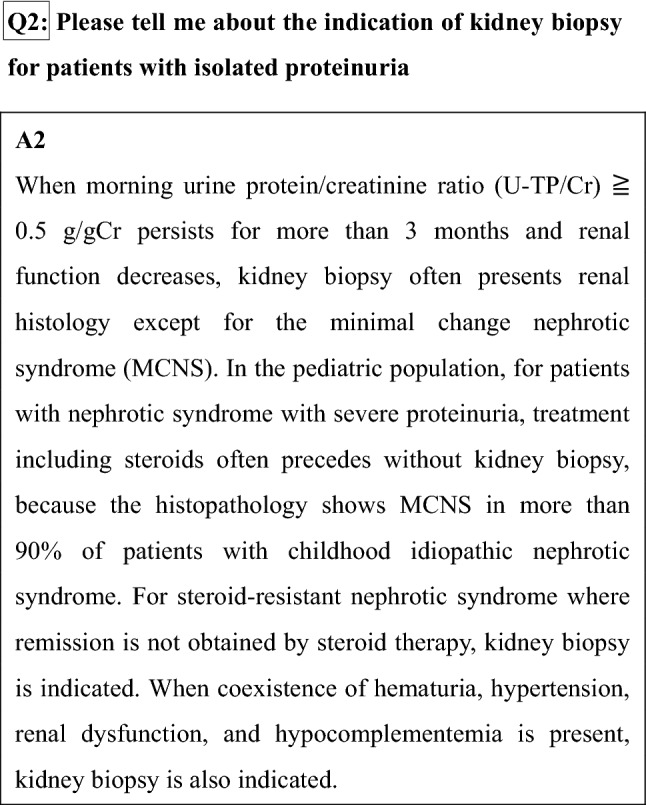



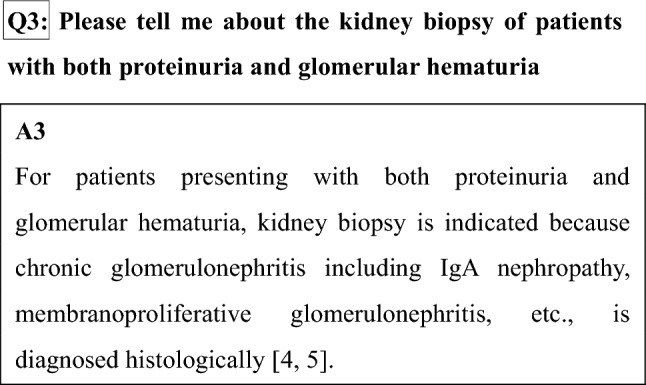



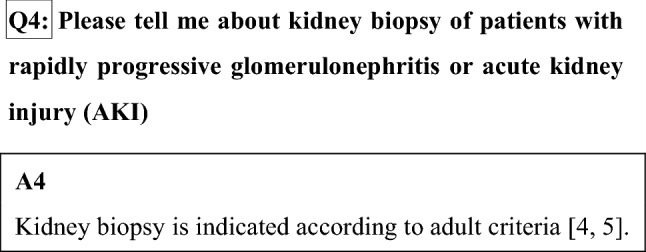



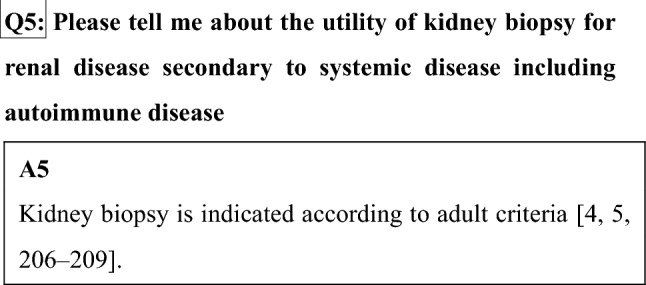



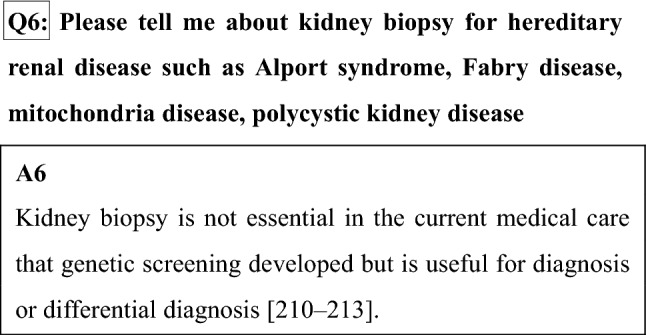


### Kidney biopsy for clinical condition with high risk

Kidney biopsy is indicated according to adult criteria [214].

### Pre-biopsy evaluation

We may need sedation or general anesthesia in children. Therefore, it is necessary to evaluate the airway and the overall status (underlying disease) beforehand. 
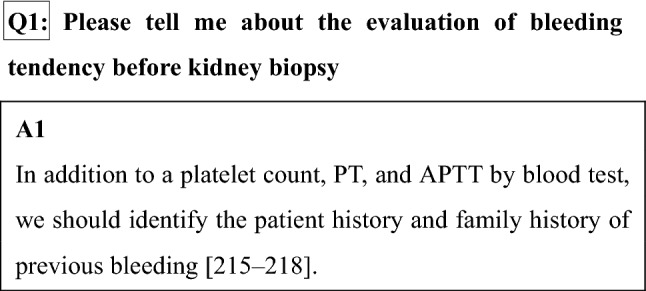



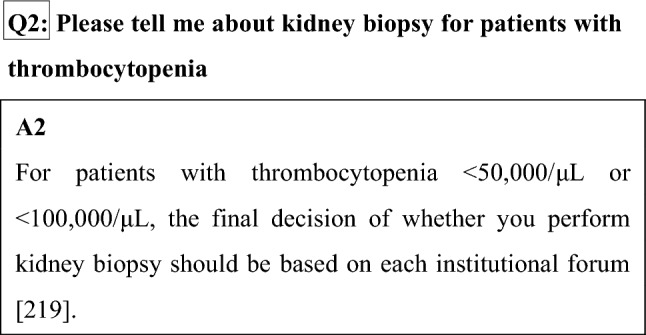



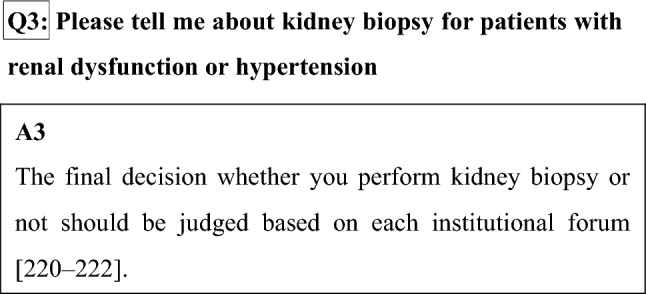


### Informed consent for kidney biopsy, explanation about kidney biopsy


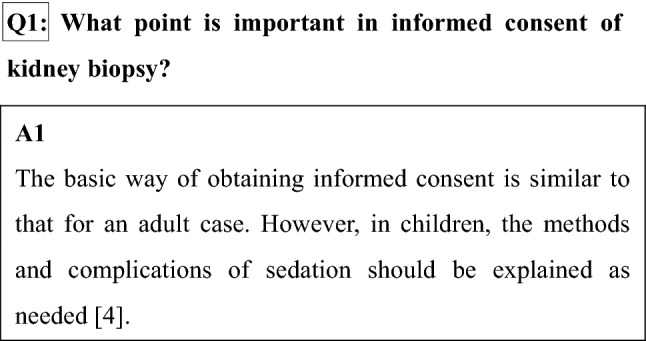



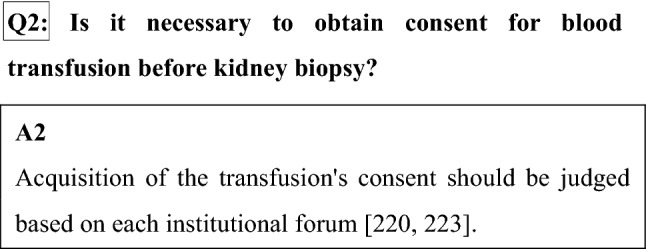



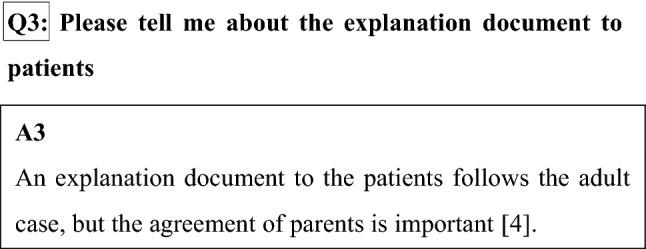


### Method of kidney biopsy (technique)


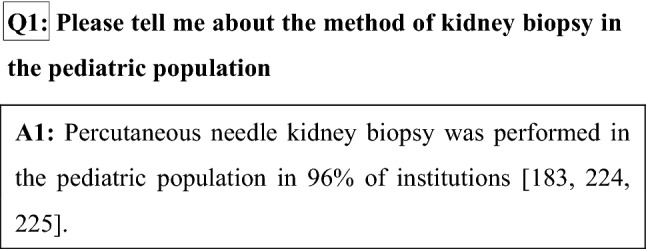



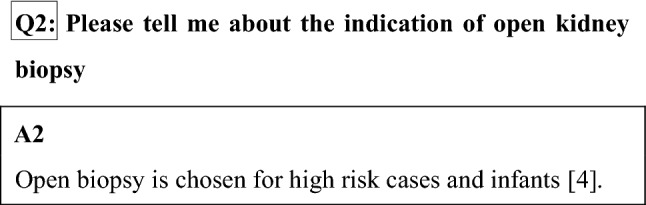



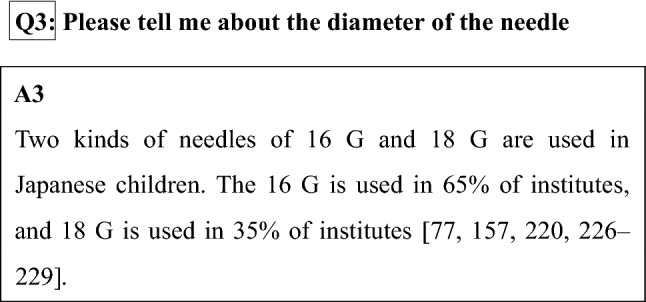


### Sedation


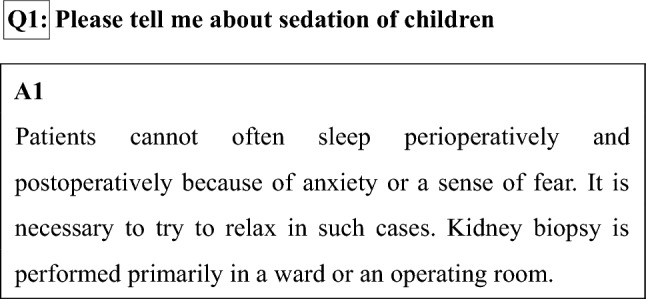



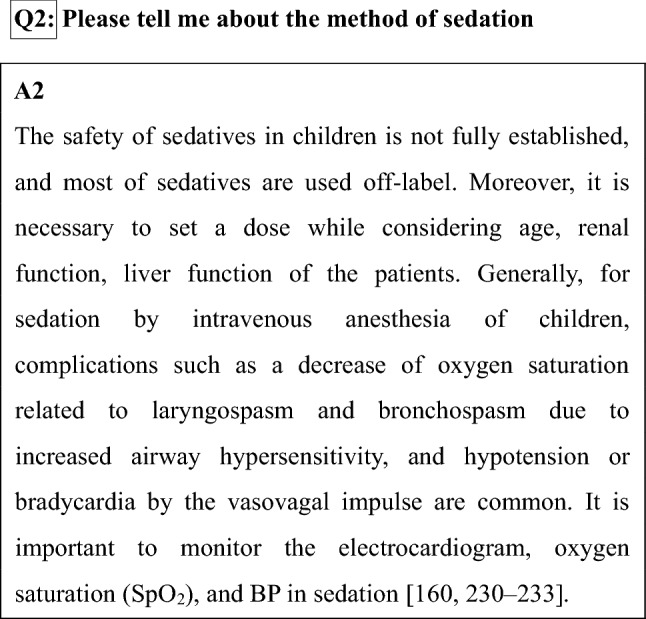



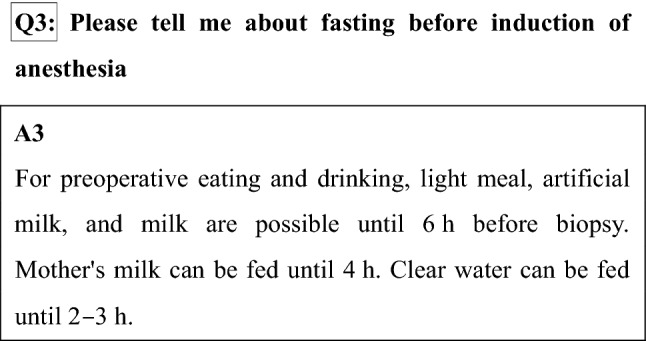



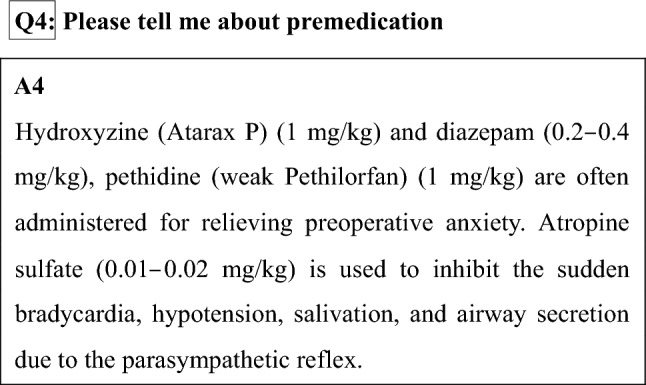



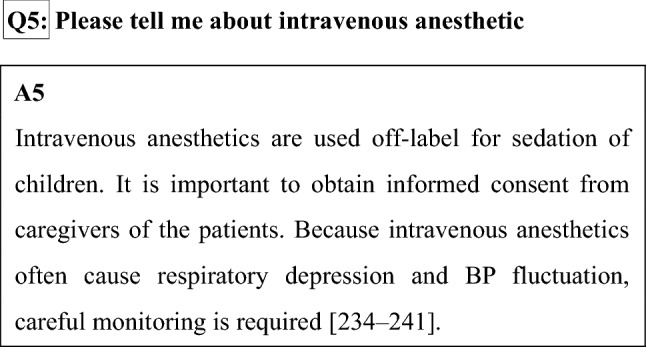

1. Ketamine (Ketalar) (initial dose, 1–2 mg/kg): duration of action is 5–10 min.
2. Midazolam (Dormicum) (initial dose, 0.05–0.1 mg/kg): half-life in blood is 0.8–1.8 h.
3. Pentazocine (Sosegon) (initial dose, 0.5–1.5 mg/kg): half-life in blood is 3–4 h
4. Thiopental (Ravonal), Thiamylal (Isozol) (initial dose, 4–6 mg/kg): duration of action is ten minutes.
5. Propofol (Diprivan) (initial dose, infants 3–5 mg/kg, older children 2.5–3 mg/kg): duration of action is 5–15 min.

### After care of the biopsy and post procedure observation


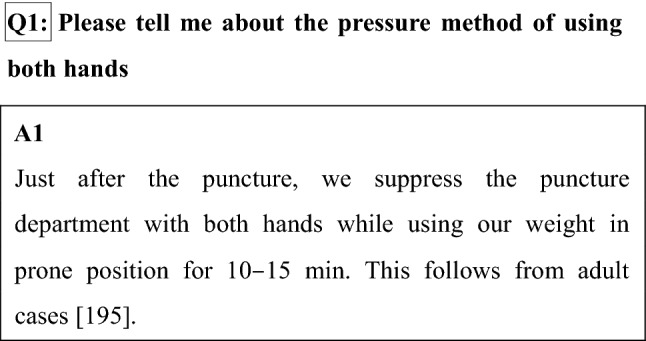



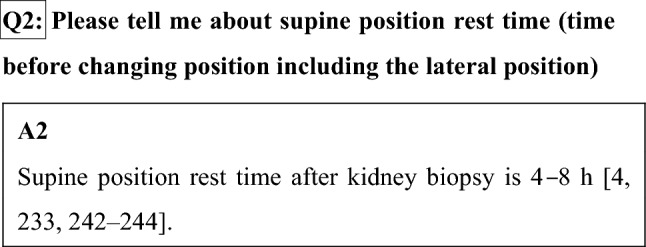
 
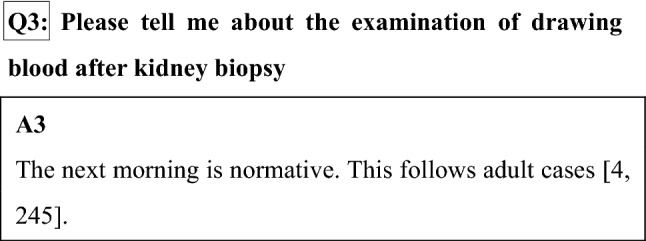



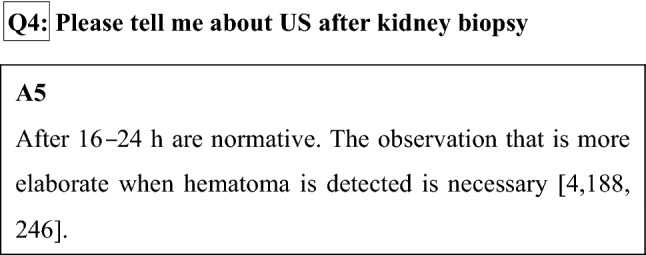



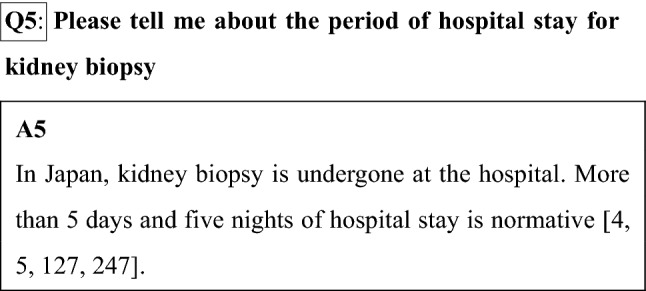


### Complications


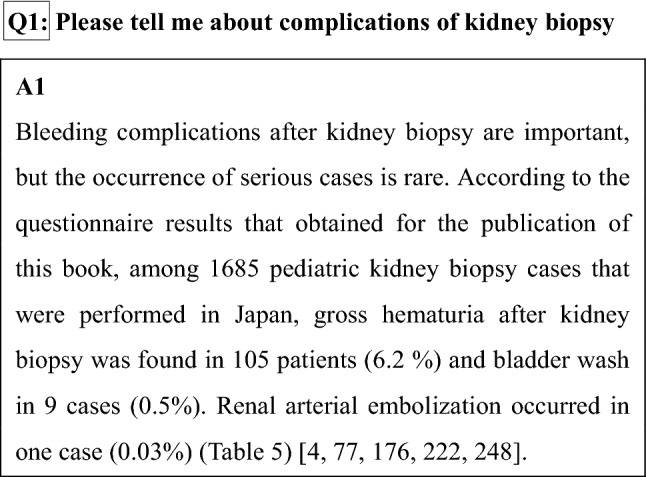


## Chapter 10: Biopsy of transplanted kidney

For the long-term engraftment after the renal transplant, early detection and early treatment for rejection or early detection of the side effect with the immunosuppressive drug are important. Because treatment totally varies according to clinical condition, the pathological evaluation of the renal graft tissue is important in treatment strategy decision. These clinical conditions occur asymptomatically and may progress.


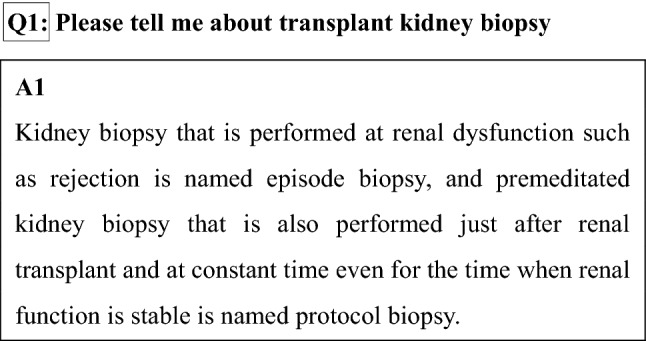

1. Episode biopsy: Transplant kidney biopsy is generally performed when an acute renal allograft rejection is suspected within a year after operation. The main clinical indicator is an increase in serum creatinine levels of 20% above a baseline value. Furthermore, a year after operation, for patients with renal dysfunction or proteinuria, the following diseases are clarified by kidney biopsy; chronic allograft nephropathy (CAN), chronic rejection (antibody-mediated rejection and T cell-mediated rejection), recurrence of underlying disease and calcineurin inhibitors nephrotoxicity [76, 149, 249].
2. Protocol biopsy: kidney biopsy is performed at the renal transplant surgery for 0 h (just after perfusion of the isolated kidney), an hour (after renal graft blood flow resumption), at post transplantation 2–3 months, and at a year after. Whether immunosuppressive therapy is appropriate, asymptomatic acute rejection occurs, or underlying disease recurs can be determined.

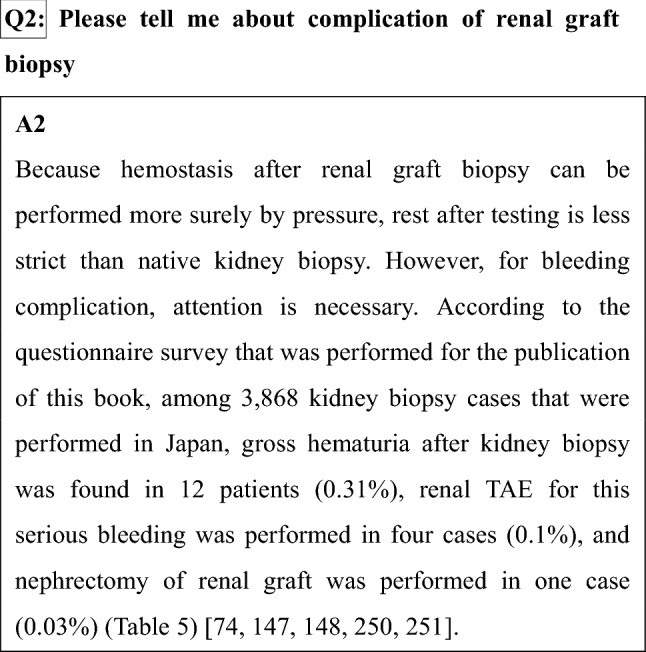



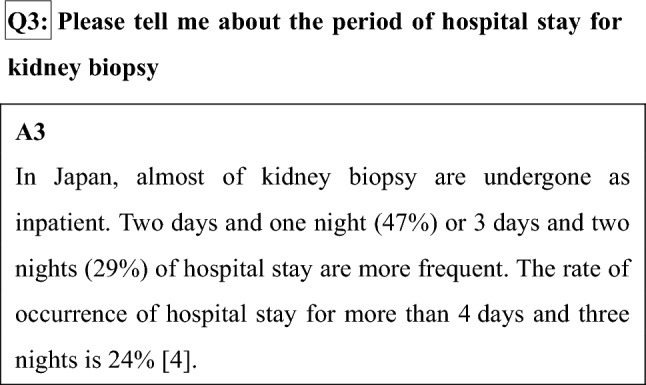


## Chapter 11: Open (surgical) kidney biopsy and laparoscopic kidney biopsy


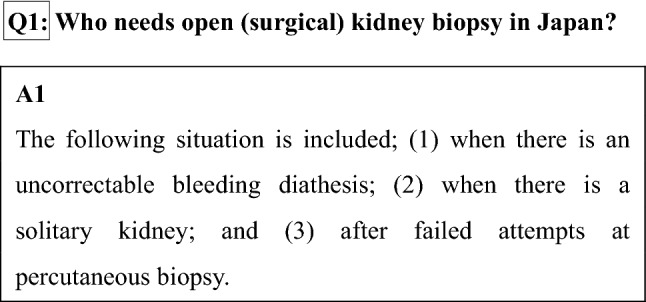


Surgeons directly look at the surface of the kidneys and determine the area from which the tissue samples should be taken. There are two type of methods including a needle biopsy and wedge biopsy. The incidence of severe bleeding of renal surface is very low, and mortality is rare, but the risk of hemorrhage into the urinary tract exists. Attention is necessary for the development of renal arteriovenous fistula (arteriovenous fistula: AVF) causing bleeding to the urinary tract. Other relatively minor postoperative complications including fever, atelectasis, and ileus can occur. In addition, an open biopsy under general anesthesia is associated with a longer hospital stay and a larger surgical scar. On wedge biopsy, the specimens may increase the proportion of shallow layer of the cortex resulting in less information of the cortex deep part and medulla [4].


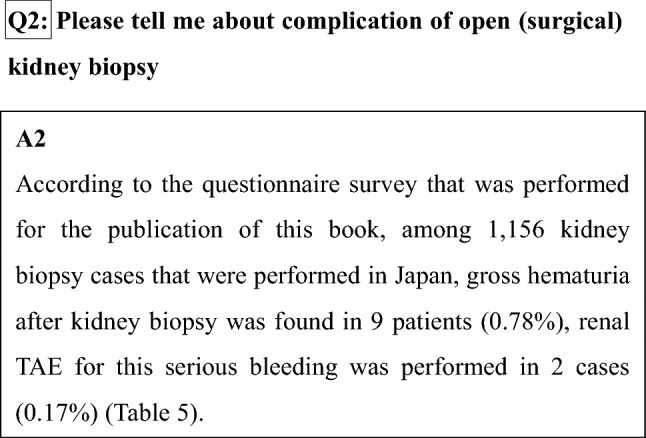



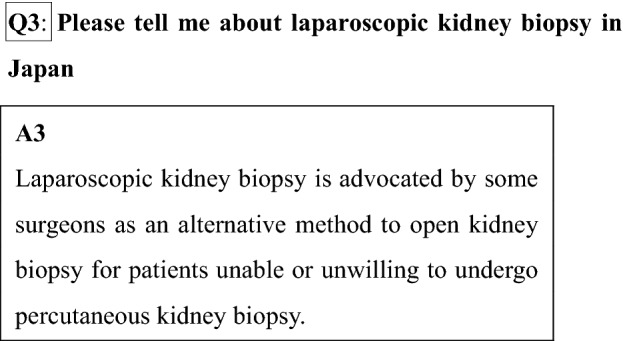

- As for the complications peculiar to laparoscopic kidney biopsy, nephric subcapsular hematoma, subcutaneous emphysema, peritoneal injury, and injury of the circumference organ are reported.
- An advantage of laparoscopic kidney biopsy in comparison with the percutaneous kidney biopsy includes certain sampling of renal tissue as well as confirmation and hemostasis of a bleeding point.
- An advantage of laparoscopic kidney biopsy in comparison to open kidney biopsy includes shortening of the hospital stay, pain reduction, and compatibility of the incised wound [4].

## Abbreviations

APTT: Activated partial thromboplastin time
ANCA: Antineutrophil cytoplasmic antibody
BP: Blood pressure
CKD: Chronic kidney disease
CT: Computed tomography
DKD: Diabetic kidney disease
ds-DNA: Double-stranded DNA antibodies
DM: Diabetes mellitus
EM: Electron microscopy
EGPA: Eosinophilic granulomatosis with polyangiitis
EB virus: Epstein–Barr virus
EBER: Epstein–Barr virus-encoded small RNA
FDP: Fibrin/fibrinogen degradation products
FSGS: Focal segmental glomerulosclerosis
GBM: Glomerular basement membrane
GPA: Granulomatosis with polyangiitis
HBV: Hepatitis B virus
HCV: Hepatitis C virus
HUS: Hemolytic-uremic syndrome
HIV: Human immunodeficiency virus
IF: Immunofluorescence microscopy
ISH: In situ hybridization
LM: Light microscopy
MRI: Magnetic resonance imaging
MPA: Microscopic polyangiitis
MN: Membranous nephropathy
MCNS: Minimal change nephrotic syndrome
NAG: *N*-Acetyl-β-d-glucosaminidase
PT: Prothrombin time
PCR: Polymerase chain reaction
RNP: Anti ribonucleoprotein antibody
SI: Selectivity index
SM: Anti-Smith (anti-Sm) antibody
SLE: Systemic lupus erythematosus
TMA: Thrombotic microangiopathy
TTP: Thrombotic thrombocytopenic purpura
TAFRO: Thrombocytopenia, anasarca, myelofibrosis, renal dysfunction, and organomegaly
US: Ultrasonography
α1MG: α1-Microglobulin
β2MG: β2-Microglobulin
